# An *In Silico* Knockout Model for Gastrointestinal Absorption Using a Systems Pharmacology Approach - Development and Application for Ketones

**DOI:** 10.1371/journal.pone.0163795

**Published:** 2016-09-29

**Authors:** Vittal Shivva, Ian G. Tucker, Stephen B. Duffull

**Affiliations:** School of Pharmacy, University of Otago, Dunedin, New Zealand; National Institute for Agronomic Research, FRANCE

## Abstract

Gastrointestinal absorption and disposition of ketones is complex. Recent work describing the pharmacokinetics (PK) of d-β-hydroxybutyrate (BHB) following oral ingestion of a ketone monoester ((*R*)-3-hydroxybutyl (*R*)-3-hydroxybutyrate) found multiple input sites, nonlinear disposition and feedback on endogenous production. In the current work, a human systems pharmacology model for gastrointestinal absorption and subsequent disposition of small molecules (monocarboxylic acids with molecular weight < 200 Da) was developed with an application to a ketone monoester. The systems model was developed by collating the information from the literature and knowledge gained from empirical population modelling of the clinical data. *In silico* knockout variants of this systems model were used to explore the mechanism of gastrointestinal absorption of ketones. The knockouts included active absorption across different regions in the gut and also a passive diffusion knockout, giving 10 gut knockouts in total. Exploration of knockout variants has suggested that there are at least three distinct regions in the gut that contribute to absorption of ketones. Passive diffusion predominates in the proximal gut and active processes contribute to the absorption of ketones in the distal gut. Low doses are predominantly absorbed from the proximal gut by passive diffusion whereas high doses are absorbed across all sites in the gut. This work has provided mechanistic insight into the absorption process of ketones, in the form of unique *in silico* knockouts that have potential for application with other therapeutics. Future studies on absorption process of ketones are suggested to substantiate findings in this study.

## Introduction

Gastrointestinal absorption of drugs is a complex process and involves various regions of the gut and transport mechanisms. Several experimental methods are available for quantification of drug transport across the gastrointestinal tract. The most commonly used *in vitro* methods include cell free models such as partial artificial membrane permeability assay (PAMPA) [1] and cell-based models such as Caco-2 permeability assay models [2]; *ex vivo* models for drug absorption include the everted sac technique [3]; and *in vivo* models use several preclinical species such as rodents, non-rodents and non-human primates [4]. While some of these techniques provide qualitative information about drug absorption (e.g., absorption properties using PAMPA and *in vitro* Caco-2 cell line) and others may quantify drug absorption (e.g., estimation of oral relative bioavailable fraction in rodents and non-rodents), the potential for exploration of mechanisms of absorption is limited with these approaches. Several modifications to these models are now available to study mechanistic aspects of drug transport. Of these, *in vitro* Caco-2 efflux transporter knockdown cell models, transfected models [5] and knockout mouse models for genes specific to intestinal transport proteins are being used [6, 7]. These methods while mechanistically important, may not directly scale to the clinic due to variability in expression of transporters that are linked with disease states in the specific cell lines and due to variability in the expression and/or function of transporters in humans in comparison to preclinical animal models [8]. Another approach involves the development of *in silico* (mathematical) mechanistic models for predicting oral drug absorption. There are proprietary models used for predictive purposes in drug development, including the ACAT model [9] implemented in GastroPlus^™^, the ADAM model [10] implemented in Symcyp^®^ and the GITA model [11]. In this work we explore an *in silico* systems pharmacology model that includes knockout variants as a means of investigating mechanisms of intestinal drug absorption. We use d-β-hydroxybutyrate (BHB) to demonstrate the approach.

BHB, acetoacetate (AcAc) and acetone are endogenous metabolites of fatty acid metabolism, produced in the liver in response to starvation. BHB and AcAc are metabolically important. Typical concentrations of total endogenous ketones in blood, under normal conditions are less than 0.5 mM. Blood ketone concentrations equivalent to starvation-induced ketosis (> 5 mM, typically achieved after a week-long fasting) may have therapeutic utility in a number of clinical conditions, specifically in treating neurological disorders [12]. Ketones have long been used in the treatment of paediatric epilepsy, with increasing evidence in the treatment of various other neurodegenerative disorders such as Alzheimer’s and Parkinson’s diseases [13, 14]. A novel method of inducing ketosis, is by consumption of a ketone monoester ((*R*)-3-hydroxybutyl (*R*)-3-hydroxybutyrate) that hydrolyses *in vivo* to produce BHB [15]. A recent population pharmacokinetic (PK) model for BHB, following oral ingestion with the ketone monoester has suggested complicated PK properties for ketones [16]. This work highlighted the potential for multiple input sites, nonlinearity associated with input and disposition of BHB and the influence of feedback inhibition processes on endogenous ketone production. It also highlighted the need to explore and understand the mechanisms of input and disposition of ketones for its development as a therapeutic agent.

Systems pharmacology provides an interface between systems biology and pharmacology [17]. Application of systems pharmacology has increased in clinical pharmacology [18] and in drug discovery and development [19]. Systems biology models are a collection of repeating modular units (such as proteins or cells or organs) where each unit has a defined set of activities. Systems biology models can be used to study spatial and temporal interactions between these modules. Thus integrating the features of systems biology in developing the *in silico* structure of the biological component and incorporating a systems pharmacology approach to incorporate the therapeutic target will provide greater opportunity in exploring the contribution and influence of therapeutics on the biological systems. In this work, we demonstrate this by combining different modules of varying activity (for transport) across the gut to generate a simplified whole human system. This model is subsequently explored to study the influence of mechanistic processes, on absorption of ketones.

The objectives of this work were 1) to develop a systems pharmacology model that represents the gastrointestinal absorption of the ketone monoester and its systemic catabolism and 2) to explore mechanistic features of gastrointestinal absorption of ketones using *in silico* knockout variants.

## Materials and Methods

### Literature search

A literature search was conducted using Medline, Embase and Cochrane libraries using defined keywords (e.g., ketones, monocarboxylate transport proteins, starvation ketosis, metabolism of ketones and kinetics of ketones) to identify and collate information on the production, transport, and disposition of ketones (both endogenous and exogenous) and the factors governing these processes. The review focussed on identifying the metabolic processes and sites of metabolism, mechanisms involved in the transport of ketones across the gastrointestinal tract and consumption in the tissues for energy production. Information on pathway of endogenous production and associated feedback mechanisms were also identified.

### General form of the mathematical framework of the systems model

The systems model is written as ordinary differential equations (ODEs) in MATLAB^®^ (ver R2014a; The MathWorks Inc., MA, US). All reaction rates are defined as moles/time (mmol/h). Reactions and transport of ketones are defined either as first-order or saturable (Michalis-Menten) processes. The general form of the equation used in this model, to represent the movement of a molecule from a given state is

$$\frac{dA_{i}}{dt}=f_{i,a}\cdot J_{i}\left(A_{i,}t\right)\cdot A_{i},$$ (1)

where *f*_*i*,*a*_ is the fractional millimoles of the *i*^th^ state undertaking the *a*^th^ process (e.g. a transport or metabolism process), *A*_*i*_ is the amount (mmol) of the *i*^th^ state. *J*_*i*_ is the flux rate (either a first-order rate constant (i.e., independent of *A*_*i*_ and *t*) or a rate constant of a saturable process (Michalis-Menten)) that is dependent on amount (mmol) at *t*^th^ time. All saturable transport processes and competitive inhibition for transport of substances was based on fractional receptor/transporter occupancy, which is similar to the Michaelis-Menten equation used to describe the saturable enzyme kinetics [20, 21]. The general form of the equilibrium rate constant for a saturable process is defined as:

$$J_{i}=V_{max,i}\cdot \left(\frac{1}{k_{m,i}+A_{i}}\right).$$ (2)

Here *V*_max,*i*_ is the maximum velocity of the saturable process for *i*^th^ state and *k*_m,*i*_ is the Michaelis-Menten constant that represents the amount (mmol) of the state where the velocity is half of the maximum. Note, all reactions/substrate transfers in the model are associated with a fraction to account for a complete molar balance. Competitive inhibitory processes for transport of *i*^th^ state (competition exerted by *j*^th^ state) are written in the form:

$$\frac{dA_{i}}{dt}=f_{i,a}\cdot V_{max,i}\left(\frac{\frac{1}{k_{m,i}}}{1+\frac{A_{i}}{k_{m,i}}+\frac{A_{j}}{k_{m,j}}}\right)\cdot A_{i},$$ (3)

where *A*_*j*_ is the amount (mmol) of *j*^th^ state and *k*_m,*j*_ (in mmol) is the Michaelis-Menten constant for the *j*^th^ state. Production of endogenous ketones is assumed to be constant with zero-order input. The negative feedback effect of circulating factors in the blood, on endogenous production is defined by a saturable process. The complete set of ODEs is provided in S1 Methods.

### Parameter values in the systems model

Values for the parameters in the system model such as rate constants for transport of substances down the gut (i.e., gastric emptying time in the fasted state, small intestinal transit, colonic transit), volumes of tissues/organs were obtained from physiological parameters in the literature [22–24] and were used in the model. Parameters for carrier mediated transport (such as *k*_*m*_) were either taken directly from the literature [25–27] or scaled (such as *V*_*max*_) from *in vitro* and/or preclinical data [25, 28, 29]. Some parameters related to passive transport (such as passive absorption from the gut) were scaled from the parameter estimates from the empirical data [16]. Remaining parameters and some sensitive parameters (identified by sensitivity analysis (see S1 Methods)) were estimated by extended least squares estimation using the *fmincon* algorithm in MATLAB^®^ (ver R2014a; The MathWorks Inc., MA, US) from the empirical data (see S1 Methods). A complete list of initial conditions of the states in each component, parameter values in the systems model can be found in S1 and S2 Tables, respectively.

### Model calibration

The model was calibrated by comparing the predictions (blood concentrations of BHB) of the systems model against empirical data at two dose levels (192 and 573 mg/kg of the ketone monoester). The data arose from a balanced design, where each individual at each dose level provided a pharmacokinetic blood sample at each time point. Since these data had several subjects per dose level the mean of the data was selected at each time point. These two doses were the extreme doses used in a clinical study [30]. The model was evaluated by visual inspection of an overlay of the model predictions of blood BHB concentration versus time over the empirical data at the two dose levels.

### Development and exploration of knockout variants

In order to explore the influence and contribution of each subcomponent of the gut and role of specific absorption processes in the gut, knockout variants for absorption were created for regions and processes in the gut (see ‘Exploration of knockout variants’ in Results section). Knockouts were created by setting an indicator variable that governs a whole process to 0 (not operational) or 1 (operational). Separate knockouts of apical and basolateral transporters were created and a ‘passive absorption knockout’ was created by eliminating passive absorption from the model. A total of 10 knockout variants were identified across the length of the gut. These knockout variants were tested either alone or in various combinations (see ‘Exploration of knockout variants’ in Results section), in order to understand the contribution and influence of specific subcomponents and processes in the gut, on the gastrointestinal absorption of ketones. The influence of processes and regions of the gut on the absorption of ketones using knockouts were studied as 1) qualitative assessment and 2) quantitative assessment.

#### Qualitative assessment of influence of knockouts

All knockout variants were tested by studying simulated blood BHB concentration-time profiles. During exploration using knockout variants, model simulations were overlaid against the empirical data and compared for deviation in the profiles. Whenever the simulated profile deviated from the empirical profile in a knockout plot, the corresponding process/region was deemed to be influential. The greater the deviation of simulations from empirical data (as shown on visual inspection), the larger the contribution of the process/region to the absorption process.

#### Quantitative assessment of influence of knockouts

The influence of knockouts on the absorption process was studied quantitatively by computing the fractional contribution of *AUC* (Area Under the Curve for BHB in blood) and shift in *T*_max_ (time to maximum concentration of BHB in the blood). Fractional *AUC* was computed as:

$$AUC_{fraction}=\left(1-\frac{AUC_{knockout}}{AUC_{full}}\right),$$ (4)

here *AUC*_*knockout*_ is the *AUC* of BHB in blood when the specific knockout was applied (Tables 1 & 2) and *AUC*_*full*_ is *AUC* of BHB in blood when no knockout was applied. A value of 1 indicates that the knockout accounted for all absorption. An *AUC*_fraction_ = 0 indicates that knockout contributed nothing. In the case of *T*_max_, a shift in *T*_max_ (*T*_max knockout_—*T*_max full_) was computed and values away from ‘0’ were deemed to be influential. These statistics were considered in a descriptive manner in this work.

**Table 1:** Knockout variants of the systems model.

Knockout variant	Process	Region	Substrate transport affected
1	Passive diffusion	upper proximal gut	Ketone monoester, butanediol and BHB
2	Passive diffusion	lower proximal gut	Ketone monoester, butanediol and BHB
3	Active absorption	Apical side of lower proximal gut	BHB
4	Active absorption	Basolateral side of lower proximal gut	BHB
5	Passive diffusion	upper distal gut	Ketone monoester, butanediol and BHB
6	Active absorption	Apical side of upper distal gut	BHB
7	Active absorption	Basolateral side of upper distal gut	BHB
8	Passive diffusion	lower distal gut	Ketone monoester, butanediol and BHB
9	Active absorption	Apical side of lower distal gut	BHB
10	Active absorption	Basolateral side of lower distal gut	BHB

**Table 2:** Combination of knockout variants explored in the systems model.

Set	Knockout variants	Process knocked out	Region
1	1	Passive diffusion	upper proximal gut
2	2	Passive diffusion	lower proximal gut
3	5	Passive diffusion	upper distal gut
4	8	Passive diffusion	lower distal gut
5	3, 4	Active absorption	lower proximal gut
6	6, 7	Active absorption	upper distal gut
7	9, 10	Active absorption	lower distal gut
8	1	Passive diffusion	upper proximal gut
9	2, 3, 4	Passive diffusion & Active absorption	lower proximal gut
10	5, 6, 7	Passive diffusion & Active absorption	upper distal gut
11	8, 9, 10	Passive diffusion & Active absorption	lower distal gut
12	1, 2, 5, 8	Passive diffusion	upper proximal gut, lower proximal gut, upper distal gut, lower distal gut
13	3, 4, 6, 7, 9, 10	Active absorption	lower proximal gut, upper distal gut, lower distal gut
14	1, 2, 3, 4	Passive diffusion & Active absorption	proximal gut
15	5, 6, 7, 8, 9, 10	Passive diffusion & Active absorption	distal gut

Note, given exploratory nature of the current work, no statistical criterion was defined either for qualitative or for quantitative assessments that would confer significance of influence.

## Results

### System pharmacology model for catabolism of the ketone monoester

**Fig 1:**
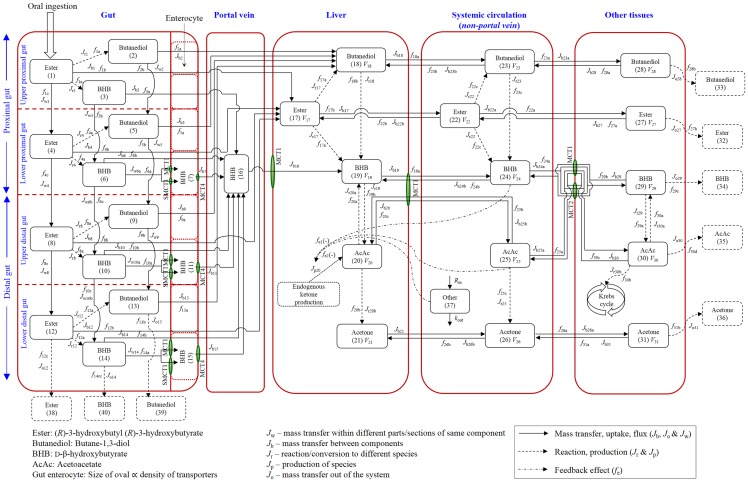
Schematic of the systems model for the absorption and catabolism of the ketone monoester following oral administration. Solid arrows represent mass transfer, uptake and flux. Dashed arrows represent reactions or productions. Dash-dot arrows represent feedback. Green ovals represent monocarboxylate (or sodium monocarboxylate) transport proteins (MCTs). State 37 denoting ‘Other’ represents the compounds such as glucose and insulin that have a feedback effect on endogenous ketogenesis.

A systems pharmacology model for catabolism of the ketone monoester and its metabolism products in humans was developed with a view to maintain the model structure to be as simple as possible but not simpler (Fig 1). The systems model was developed by integrating the information from the literature specifying the metabolic pathways, transporters involved in the flux of ketones and the knowledge gained from the empirical model-building. This systems model incorporated microscopic processes such as carrier-mediated transport at the cellular level and also the network of processes occurring at tissue/organ level accounting for the human system as a whole. The final model consists of 5 components, 40 states and 173 parameters. The components of the model include 1) luminal sites of the gut with variability in the expression of transport proteins across the sites, 2) the portal system facilitating the transport of ketones from gut to the liver, 3) the liver as the site of metabolism, site for endogenous ketone production and as a link between the portal system and systemic circulation, 4) the systemic circulation as the principal site for transport of ketones to all other tissues, site of metabolism and site for factors exerting the feedback effect on endogenous production of ketones and 5) all other lumped tissues that either consume ketones for energy production or those involved in excretion.

### Components of the systems model

A brief description of each component in the model with an emphasis on its contribution to the kinetics of ketones is outlined below.

#### Gut

The gut is divided into two subcomponents namely proximal gut and distal gut. Each of these subcomponents are further divided (upper and lower proximal gut, upper and lower distal gut), providing a representation of spatial and temporal transport (Fig 1). These regions are designed to nominally represent the stomach, proximal small intestine, distal small intestine and colon, however this representation is for convenience and should not be implied to be anatomically correct. The mechanistic basis for this division was the expression of monocarboxylate transport proteins (MCTs) that contribute to the transport of ketones across the gut wall [31, 32]. Three subtypes of MCTs namely MCT1, MCT4 (SLC16A sub-family) and SMCT1 (Na^+^ coupled MCT1, belonging to SLC5A8 sub-family) are expressed along the gut wall. Of these, MCT1 and SMCT1 are expressed on the apical side and MCT4 is expressed on the basolateral side [33, 34]. These transporters vary widely, in terms of their affinity and capacity for transport of ketones; whereas MCT1 and MCT4 are low affinity and high capacity transporters, SMCT1 is a high affinity and low capacity transporter [35]. In addition to the anatomical expression and functional regulation, the proportion of expression of these proteins varies along the gut. Protein expression of these transporters (i.e., mg of protein/cm^2^) is reported to increase from proximal to distal regions of the gut [33]. This variability in expression of transport proteins along gut regions leads to variability in the capacity (expressed in terms of increased V_max_, down the gut regions in the model) of the transport of ketones. This forms the basis for four distinct subcomponents of the gut in this systems model. A diagrammatic representation of the transport proteins expression in the gut wall (enterocyte) is presented in S1 Fig. Hydrolysis of ketone ester to butane-1,3-diol (butanediol) and BHB is shown to occur along the length of the gut and esterases and carboxylesterases expressed throughout the gut wall facilitate this hydrolysis [36]. Transport of ester and butanediol is a passive process whereas both active and passive processes contribute to transport of BHB across the gut wall. Compounds that are not absorbed from the gut are excreted out in the faeces.

#### Portal vein

The portal vein forms the link between gut and the liver. Ketones absorbed from across the gut wall are taken by the portal system and transported to the liver.

#### Liver

The liver forms the major connecting link for absorption of ketones from the gut to the systemic circulation. The liver is represented as the site of metabolism and the site of endogenous ketone production [37]. Various metabolic processes such as hydrolysis of the ketone ester to butanediol and BHB, hydrolysis of butanediol to BHB, interconversion of BHB and AcAc and irreversible decarboxylation of AcAc to acetone are considered to take place in the liver [38]. Endogenous ketones are produced from oxidation of fatty acids in the liver to form AcAc [39]. Further conversion of AcAc to other ketones in the liver is as shown in the Fig 1. Transport of BHB and AcAc into systemic circulation is mediated by MCT1 proteins [35]. All other compounds are assumed to be transported in and out of the liver by passive diffusion process.

#### Systemic circulation

The systemic circulation is the site for transport of ketones in the body and also a site of metabolism of ketone ester to butanediol and BHB, butanediol to BHB and AcAc to acetone [36]. All other compounds (endogenous and exogenous) in the blood, other than ketones, that exert a feedback effect on endogenous ketogenesis are represented as a single lumped state (state # 37 in Fig 1). Transport of BHB and AcAc between systemic circulation and liver is mediated by MCT1 [35] and between all other tissues by MCT1 and MCT2 [26, 35, 40]. All other compounds are distributed in and out of the systemic circulation by passive diffusion.

#### Other tissues

All other organs and tissues into which ketones are transported from the systemic circulation are presented together as one lumped component. This constitutes sites of metabolism such as heart, brain, kidney and skeletal muscle and sites of excretion such as lungs and kidneys. Transport of ketones (BHB and AcAc) in and out of the tissues from the systemic circulation is an active process and is facilitated by MCT1 and MCT2 transporters. MCT2 belongs to the SLC16A family of transporters and is a high affinity and low capacity transporter for both BHB and AcAc [29]. All other compounds are transported in and out of tissues by passive diffusion process. Within the tissues, BHB and AcAc are converted to acetyl Co-A, and used in energy production via the Krebs cycle. Ketones that are neither used in energy production nor redistributed into blood, are assumed excreted either from the kidneys or in the form of acetone expelled from the lungs [41, 42].

### Calibration of the systems model

**Fig 2:**
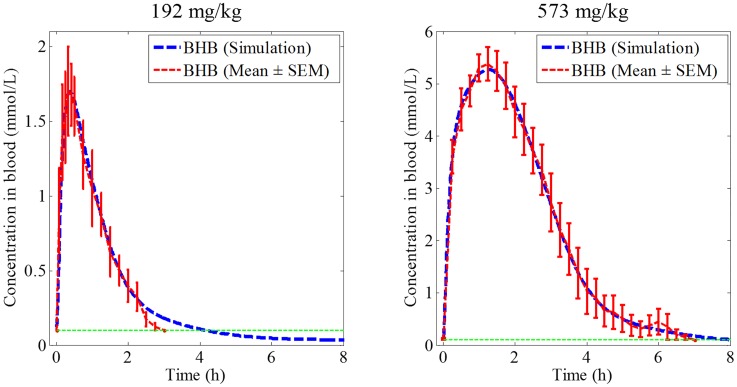
A single deterministic simulated time course of blood BHB concentrations. Simulations (blue dotted line) overlaid on empirical data (red dashed lines; Mean ± SEM) at two dose levels (192 mg/kg and 573 mg/kg) of the ketone monoester. The dashed green line at bottom of each graph represents lower limit of quantification of BHB in blood.

Given the objectives of the current work to explore mechanisms of gastrointestinal absorption of ketones, a fit-for-purpose approach was used in calibrating the systems model as opposed to routine model evaluation tests used for standard PKPD models [43, 44]. The model provided a good description of the blood BHB profiles with simulations at both dose levels being within the mean ± Standard Error of the Mean (SEM) of the empirical data for most of the data points for both dose levels (Fig 2). The final set of parameter values are provided in S2 Table.

### Exploration of knockout variants

Influence and contribution of processes and regions of the gut on the absorption of ketones was studied using *in slico* knockout variants. Assessment of influence using knockouts were studied both qualitatively and quantitatively.

#### Qualitative assessment of influence of knockouts

**Fig 3:**
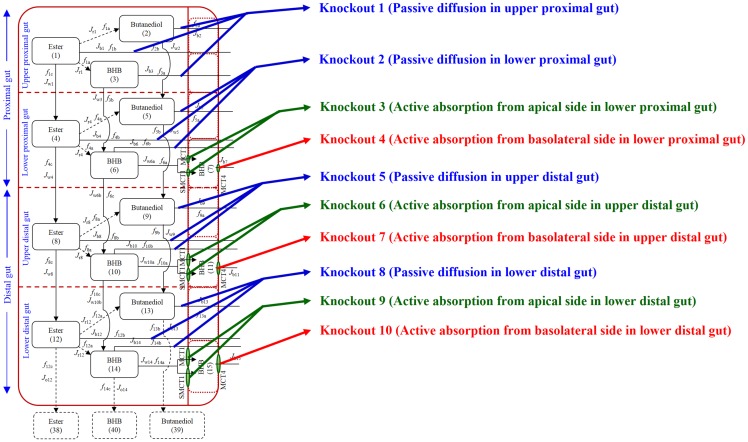
Schematic representation of 10 knockout variants used in the systems model. Knockouts shown in blue account for passive diffusion, those shown in green account for active absorption from apical side and those shown in red account for active absorption from basolateral side in the gut. In case of knockout 1 passive diffusion in the upper proximal gut was knocked out, in knockout 3 active absorption on the apical side of the lower proximal gut was knocked out and in knockout 4 active absorption on the basolateral side of the lower proximal gut was knocked out.

**Fig 4:**
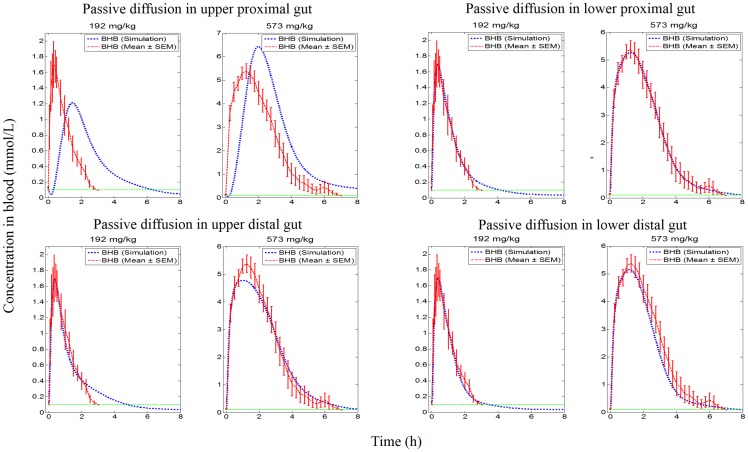
Simulation profiles of BHB in blood against empirical data at two dose levels for knockout of passive diffusion along subcomponents of the gut.

**Fig 5:**
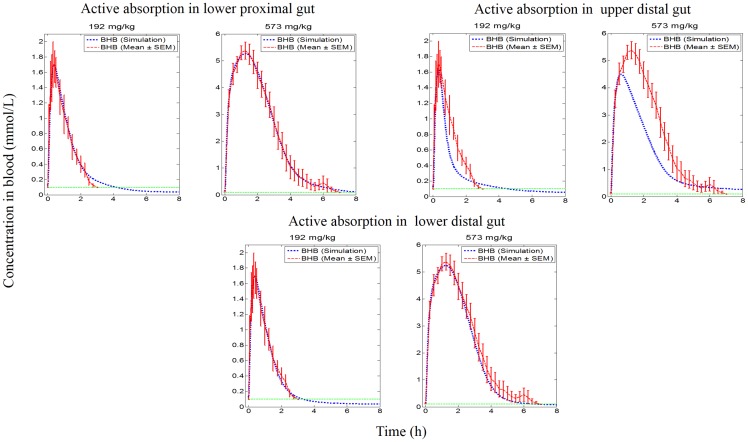
Simulation profiles of BHB in blood against empirical data at two dose levels for knockout of active absorption process along subcomponents of the gut.

Knockout variants of the systems model (see Fig 3 and Table 1) were explored in the order specified in Table 2. Knockout of passive diffusion in each sub-component of the gut (knockout sets 1, 2, 3 and 4 in Table 2) indicated that passive diffusion from the upper proximal gut was a significant contributor for absorption at both dose levels. The contribution of passive diffusion in the lower proximal gut and upper distal gut appeared to be minimal for both dose levels. However, contribution of passive diffusion in the lower distal gut was minimal for the low dose but was important for the high dose level (see Fig 4). Exploration of active absorption processes, by knocking out MCTs on both apical and basolateral sides (knockout sets 5, 6 and 7 in Table 2), showed that active transport in the lower proximal gut was not important at either dose levels, but the upper distal gut was found to play an important role at both dose levels tested. The lower distal gut was influential in the absorption of ketones via active absorption at the high dose level but had a minimal contribution for absorption at the low dose level (see Fig 5).

**Fig 6:**
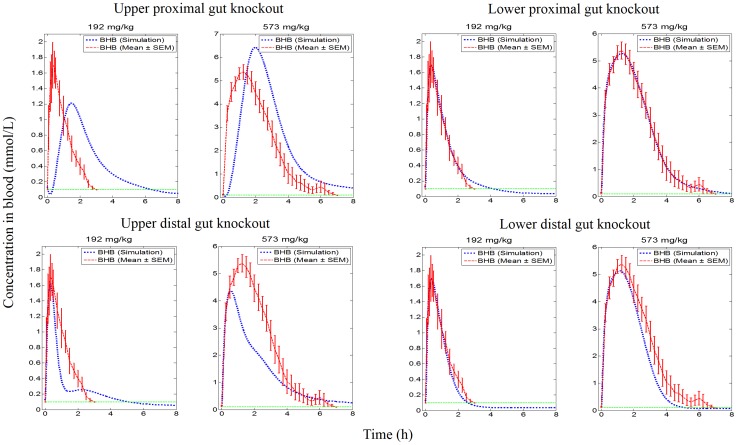
Simulation profiles of BHB in blood against empirical data at two dose levels for knockout of each sub-component (passive and active transport knockout) of the gut.

**Fig 7:**
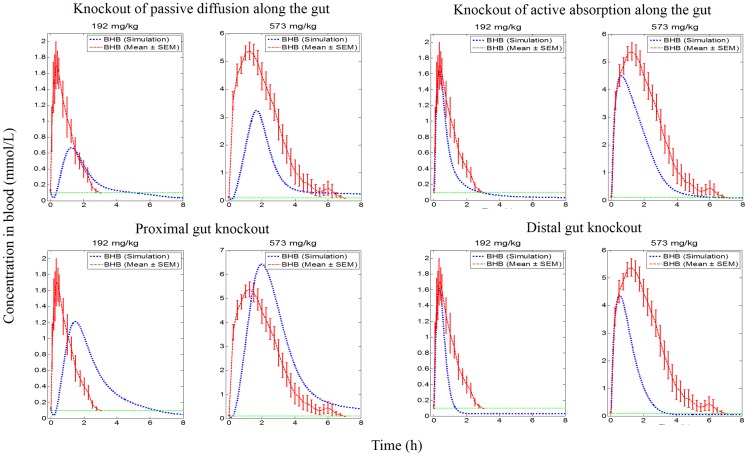
Simulation profiles of BHB in blood against empirical data at two dose levels for knockout of regions and the processes in the gut. Knockout of processes (passive and active transport) along the length of the gut (upper panel) and knockout of regions (proximal and distal) of the gut (lower panel) at two doses.

Knockout of all absorption processes in successive subcomponents of the gut (knockout sets 8, 9, 10 and 11 in Table 2), indicated that the upper proximal gut contributed the most for both dose levels tested. The contribution of the lower proximal gut was found to be less important. In the case of the upper and lower distal gut regions, their contributions were found to be important at the high dose level but this was negligible at the low dose level (see Fig 6). Knockout of passive diffusion across the length of the gut (knockout set 12 in Table 2) indicated that this process was a major contributor for both dose levels. Knockout of active absorption across the length of the gut (knockout set 13 in Table 2) indicated that its fractional contribution was very important for the high dose level whereas it was marginal at low dose. Knockout of the proximal gut (knockout set 14 in Table 2; knock out of all absorption processes in upper and lower proximal gut) indicated that this region contributed greatly for absorption of ketones at both dose levels. Knockout of distal gut (knockout set 15 in Table 2; knock out all absorption processes in upper and lower distal gut) indicated that contribution of this region to absorption of ketones was minimal at the low dose but more substantial at the high dose level (Fig 7).

#### Quantitative assessment of influence of knockouts

Results from exploration of quantitative influence of knockouts (*AUC*_*fraction*_ and shift in *T*_*max*_) are presented in Table 3. These results are in agreement with observations from qualitative assessment. Quantitatively, three potential sites contributed to absorption of ketones. Passive diffusion had a greater contribution in the proximal gut and active absorption played a major role in the distal gut. Although highly influential (based on shift in *T*_*max*_), a negative *AUC*_*fraction*_ was observed for knockout of passive diffusion (knockout set 1, 8 and 14 in Table 3) in the upper proximal gut. This can be attributed to the nature of first-order process assumed for transport down across the gut regions and passive absorption processes in other regions of the gut.

**Table 3:** Quantitative assessment of influence of knockouts in the systems model.

Set	Knockout variants	*AUC*_*fraction*_		Shift in *T*_*max*_ (h)	
		192 mg/kg	573 mg/kg	192 mg/kg	573 mg/kg
1	1	0.01	-0.14	1.09	0.72
2	2	0.00	0.00	0.00	0.00
3	5	0.06	0.01	-0.02	-0.22
4	8	0.07	0.11	0.00	-0.10
5	3, 4	0.00	0.00	0.00	0.00
6	6, 7	0.29	0.31	-0.04	-0.64
7	9, 10	0.05	0.08	0.00	-0.03
8	1	0.01	-0.14	1.09	0.72
9	2, 3, 4	0.00	0.00	0.00	0.00
10	5, 6, 7	0.33	0.33	-0.05	-0.71
11	8, 9, 10	0.11	0.18	0.00	-0.13
12	1, 2, 5, 8	0.49	0.57	0.89	0.41
13	3, 4, 6, 7, 9, 10	0.34	0.40	-0.04	-0.65
14	1, 2, 3, 4	0.01	-0.14	1.09	0.72
15	5, 6, 7, 8, 9, 10	0.50	0.60	-0.05	-0.72

## Discussion

We have developed a systems pharmacology model for absorption and catabolism of a ketone monoester in humans. The mathematical model is based on known mechanisms from the literature and knowledge gained from the empirical model building for BHB. In this work we introduce the concept of *in silico* knockout variants which are easily invoked in mathematical models. Model building was achieved in multiple steps following collation of information from the literature related to biochemical pathways of ketones in the body, transporters involved in their flux, endogenous ketone production, factors affecting the endogenous production, role and regulation of processes of metabolic consumption of ketones in the body (S2 Fig). Knowledge gained from the empirical model building for BHB was also helpful in developing the systems model [16]. A parsimonious model was designed, while retaining the ability to fulfil objectives of the current work. Assumptions made in developing the systems model are provided in S1 Methods.

A mechanism-driven (bottom-up) approach was used in developing the model. The design of subcomponents for the gut was driven by variability in expression of transport proteins along the gut [33, 34]. Though we have shown here four subcomponents for the gut which might reflect stomach + duodenum, jejunum, ileum and colon respectively, these assignments were not formally made.

Keeping in view, the objectives of the current work, i.e., to explore mechanisms related to gastrointestinal absorption, using of ketones as an example, a fit-for-purpose approach was used in calibrating the model [45]. This means, testing the current model with the given set of parameters (and values) for its ability to describe the mean empirical data for BHB at the two desired dose levels tested. Further evaluations were not considered for evaluating the models performance in this work. This differs from the more complicated model evaluation techniques used for data-driven empirical models that use a top down approach. The current work lends itself to an exploratory process to gain an understanding of the mechanisms of gastrointestinal absorption of ketones, from which further conjectures and hypotheses can be generated in relation to the absorption and disposition of ketones. Since model is exploratory in nature, a statistical analysis was not included in the calibration of the model. Model performance was calibrated heuristically by the visual inspection of the model predictions in comparison to the data at two dose levels.

Interpretation of results from application of knockout variants of the systems model have shown that there are at least three distinct regions in the gut that contribute to gastrointestinal absorption of ketones. Future clinical studies of absorption of ketones are recommended to test this hypothesis. We believe that the learnings from knock-outs in this model can be tested by delivery of test substance directly into duodenum, conducting clinical absorption studies in special population such as ileostomy and colectomy patients. Exploration of knockout variants have indicated that different mechanisms dominate the input process of ketones in different regions of the gut leading to dose-dependent nonlinear absorption processes. It was also observed that the low dose was predominantly absorbed from the proximal gut and that this was mainly driven by passive processes. In contrast to this, the high dose was absorbed across the length of the gut and this was predominantly driven by passive diffusion process in the proximal gut and active transport processes in the distal gut. This work highlights that while passive diffusion contributes to the initial input profiles of blood BHB, active transport processes contribute to later input processes. The findings from this work are in line with and support the findings from our previous work in which we developed an empirical population PK model for blood BHB concentration, where putative multiple absorption sites were suggested in the modelling process. The current work leads to the hypothesis that there are three distinct input sites for absorption of ketones in the gut. This hypothesis may be the subject of interest for testing in future studies with the ketone monoester, notably when particular targets for plasma profiles and exposure are defined.

Though the current systems pharmacology model for ketones was able to serve the purpose of current objectives, it is by no means complete. Exploration and application of the systems model in the current work was limited to considering the influence of absorption of the ketone monoester on blood BHB concentrations only, exploration of other ketones in blood and ketones in other tissues was beyond the scope and objectives of the current work. The current set of parameter values of the model, though providing a good description of BHB profiles in the blood, are generally not identifiable and will need to be calibrated in future work when other ketones are tested in blood and/or other tissues. The immediate areas of expansion for the current model include exploration of BHB kinetics in tissues of metabolic interest such as skeletal muscle, kidney, brain and heart and sites of excretion such as kidneys and lungs. This can be achieved by differentiation of the tissue component of the model to yield organs of interest as separate components. This may be followed by extending the scope of this model to explore kinetics of other ketones and their precursors to understand the catabolism of the ketone monoester in a broader sense. These expansions will help to provide the capability to link pharmacodynamics of ketones with the pharmacokinetics, for future prospects with the ketone monoester that are intended for therapeutic applications.

This work demonstrates the utility of the systems pharmacology model approach in exploring the mechanisms of gastrointestinal absorption of therapeutic compounds. A unique feature of this work is the development of *in silico* knockout variants in exploring the mechanism of oral drug absorption. The initial part of this work relating to model development demonstrates how processes can be integrated to build a systems pharmacology model based on known mechanisms. The later part describes how some selective processes can then be differentiated from the whole (such as the use of knockout variants shown here) to explore mechanisms that are either difficult or impossible to perform experimentally (e.g. knockout of passive diffusion, knockout of specific regions in the gut). This work demonstrates the potential of systems models to complement or, in some circumstances, replace animal studies.

In conclusion, a systems pharmacology model for the absorption and catabolism of a ketone monoester is developed. This model is capable of describing blood BHB concentrations at two dose levels. Knockout variants of components of the systems model provided a notable and convincing mechanistic insight into the gastrointestinal absorption of ketones in terms of the influence of specific regions, processes of absorption and the dose-dependent nature of absorption. The current model provides an initial platform for continuous exploration of mechanisms of ketone disposition and its further expansion to provide insight into the mechanisms of pharmacological action of ketones in therapeutic applications. This approach can be extended to other therapeutic compounds with known complexities of drug actions.

## Supporting Information

S1 Fig Schematic of anatomical expression of MCTs in the gut enterocytes. (DOCX)

S2 Fig Schematic representation of biochemical pathway of ketones *in vivo*. (DOCX)

S1 Methods Assumptions made in developing the systems model, parameter estimation in the systems model and ordinary differential equations of the systems model. (DOCX)

S1 Table Initial condition of states in the systems model. (DOCX)

S2 Table Parameter values in the systems model. (DOCX)
