# Supplementary material for: An In Silico Knockout Model for Gastrointestinal Absorption Using a Systems Pharmacology Approach - Development and Application for Ketones
Source: PLoS One. 2016 Sep 29;11(9):e0163795. doi: 10.1371/journal.pone.0163795 (PMC5042539; doi:10.1371/journal.pone.0163795)
Supplement: S1 Fig — (DOCX) [file pone.0163795.s001.docx]

**S1 Fig.**

**S1 Fig.** **Schematic showing anatomical expression of MCTs in the gut enterocytes.** MCT1 and SMCT1 are expressed on the apical side and MCT4 is expressed on the basolateral side. The level of expression of these transport proteins (mg of protein/cm^2^) increases along the length of the gut from proximal to distal end as shown by increase in the size of the ovals down the gut. This was represented in the model by an increase in *V*_max_ across the regions of the gut from proximal to distal end.
