# Supplementary material for: An In Silico Knockout Model for Gastrointestinal Absorption Using a Systems Pharmacology Approach - Development and Application for Ketones
Source: PLoS One. 2016 Sep 29;11(9):e0163795. doi: 10.1371/journal.pone.0163795 (PMC5042539; doi:10.1371/journal.pone.0163795)
Supplement: S2 Fig — (DOCX) [file pone.0163795.s002.docx]

**S2 Fig.**

**S2 Fig. Schematic representation of biochemical pathway of ketones *in vivo*.** Ketone monoester following oral ingestion, undergoes sequential hydrolysis to produce ketones. This hydrolysis take place in various regions of the body and multiple enzymes contribute to this process. Fatty acids are the major contributor to the production of endogenous ketones. Ketones produced in the liver are released into the blood stream and are taken up by extra hepatic tissues such as brain, heart, kidney and skeletal muscle and are used in the Krebs cycle for energy production.
