# Supplementary material for: An In Silico Knockout Model for Gastrointestinal Absorption Using a Systems Pharmacology Approach - Development and Application for Ketones
Source: PLoS One. 2016 Sep 29;11(9):e0163795. doi: 10.1371/journal.pone.0163795 (PMC5042539; doi:10.1371/journal.pone.0163795)
Supplement: S1 Methods — (DOCX) [file pone.0163795.s003.docx]

**SUPPORTING INFORMATION**

**An *in silico* knockout model for gastrointestinal absorption using a systems pharmacology approach - development and application for ketones**

**Assumptions made in developing the systems pharmacology model for catabolism of the ketone monoester**

1. The model incorporates components that are considered to be critical in influencing the pharmacokinetics (PK) of ketones. The gut components were identified as upper proximal, lower proximal, upper distal, lower distal. The parameter values associated with transporters in these regions were based on their resemblance to the stomach, upper small intestine, distal small intestine and colon, respectively. Despite this resemblance, these regions on the model do not necessarily hold physiological and anatomical meaning.
2. The following assumptions were made:
   1. Ester, butanediol and acetone are transported by passive processes only.
   2. In respect of parsimony, intracellular metabolic interconversion of BHB and AcAc [1, 2] was assumed not to occur in the enterocytes of the gut.
   3. Transport of BHB from the enterocytes to the gut lumen, and from the liver to the portal vein (for BHB and AcAc) was not included in the model
   4. Intracellular transporter mediated process (from cytoplasm to inner membrane of mitochondria) via MCTs for BHB and AcAc was not included in the model
   5. Transport of ketones (BHB and AcAc) in and out of red blood cells (mediated by MCTs) within the systemic circulation [3] was not included in the model
   6. Competitive inhibition for transport of exogenously administered ketones by endogenous substrates was not included in the model as the concentration differences between endogenous substrates and exogenous ketones are in the order of 5 to 10 fold.
   7. Whenever more than one compound is being transported by one transport protein a competitive model for their transport was assumed.
   8. Passive diffusion for transport of BHB and AcAc across organs (other than gut) was not included in the model as greater than 99.9% of BHB and AcAc is expected to be ionised at the physiological pH (7.4) based on their pKa (4.41 for BHB and 3.58 for AcAc) and logP values (-1.14 for BHB and -0.52 for AcAc) and the fractional contribution of passive diffusion was expected to be minimal [4].
   9. Plasma protein binding of BHB and AcAc is not reported in the literature. As ketones are reported to be ionised almost completely (> 99.9%) at physiological pH (7.4) [4], the effect protein binding of ketones was not included in the model.
3. Negative feedback effect on endogenous ketone production was expressed in the model in terms of two pathways (1) an effect mediated by exogenous ketones in blood, mediated via PUMA-G receptors [5] and (2) an effect caused by all other compounds (such as insulin, glucagon and glucose) in the circulation [6]
4. Enzymatic processes such as hydrolysis of ester, oxidation of butanediol and BHB, reduction of AcAc (to BHB) and its irreversible breakdown to acetone were assumed to be first-order, because
   1. information in the literature on specific isoforms of esterases (and their activity) in human gut, liver and blood that are responsible for the hydrolysis of ester was not available.
   2. similarly, no information was available on the activity of alcohol dehydrogenase and aldehyde dehydrogenase in metabolism of butanediol and 3-hydroxybutanal. Based on literature data related to metabolism of alcohol, several isoforms of these enzymes exist in various organs and most of these isoforms are low affinity and high capacity enzymes for alcohol metabolism.
   3. this was also the case with BHB dehydrogenase and AcAc decarboxylase enzymes. Some reports indicate that breakdown of AcAc to acetone can also be a spontaneous and non-enzymatic process [7].

**Sensitivity analysis and Parameter estimation:**

A small number of parameters in this systems model were estimated (see S2 Table) in order to calibrate the model. Prior to the parameter estimation, a sensitivity analysis was performed using complex step differentiation (in MATLAB^®^) to identify parameters that were sensitive to the absorption process.

***Sensitivity analysis:***

1. Univariate local sensitivity analysis was performed using complex step differentiation (step size = 10^-5^) to identify sensitive parameters that were required to be estimated.
2. Absolute sensitivity of parameters over time was plotted (see **Fig. S3** below) to determine sensitivity of parameters as most sensitive, moderately sensitive and less sensitive. Parameters were arbitrarily defined into anyone of the sensitivity class based on visual inspection and no statistical criterion was used.
3. Based on sensitivity analysis, parameters identified as most sensitive were f1a, f1b, f4b, f19b, f24b, f29b and f30c
4. Parameters that were moderately sensitive were f1c, f8b, f13a, f18a, f19a, f24a, f29a and f29c
5. Other less sensitive parameters influencing the model predictions were f4a, f5a, f6b, f9a, f10, f10c, f14b, f17b, f20b, f20c, Imax1, f22a, f22c, f23a and f30d

***Parameter estimation:***

1. Parameter estimation was done using fmincon algorithm in MATLAB^®^. Extended least squares objective function (*OBJ_ELS_*) was used as minimisation function in estimating the parameters

$OBJ_{ELS}=\sum_{j=1}^{n} \left[\frac{\left(y_{j}-f_{j}\right)^{2}}{\sigma_{j}^{2}}+\ln\left(\sigma_{j}^{2}\right)\right]$ Equation S1

where *n* is number of observations, *y_j_* is *j*^th^ observed data, *f_j_* is the model prediction at *j*^th^ point and *σ_j_*^2^ is the random noise in model prediction for the *j*^th^ observation.

1. Diagonal elements (noise) of variance matrix were computed using the equation below

$\sigma_{j}^{2}=\sigma_{prop}^{2}\cdot f_{j}^{2}+\sigma_{add}^{2}$ Equation S2

Where *σ^2^_prop_* is variance of proportional error and *σ^2^_add_* is variance of additive error in the *j*^th^ observation of the predicted data

1. This error model is of the form (combined error model)

$y_{j}=f_{j}\cdot\left(1+\varepsilon_{prop,j}\right)+\varepsilon_{add,j}$ Equation S3

1. The most sensitive parameters were estimated in the first instance, starting with one parameter and adding a parameter at a time in the estimation. A drop in the objective function value was considered significant (see figures 1 to 5). Estimation proceeded until a good representation of the model predictions to the empirical data was found.
2. Following estimation of most sensitive parameters, addition of moderately sensitive parameters in the estimation set does not always led to improvement in the fit as shown by OBJV (see figures 6 to 10).
3. Rate constants associated with some sensitive fractions were chosen selectively for estimation (rather than estimating the sensitive fractions as they did not improve model fit probably due to their boundary conditions between 0 to 1). They were mostly first-order processes and this change had led to a good improvement in the fit and significant drop in the OBJV (see figures 11 to 14).
4. Estimation of few parameters of carrier mediated transport (such as *V*_max_ of BHB for MCT1 in the liver and tissues) have led further drop in the OBJV and greater improvement in the fit (see figure 15).
5. Inclusion of some less sensitive parameter in the estimation too, have improved the fit and a drop in the OBJV (see figure 16).
6. Finally addition of *V*_max_ of BHB for MCT1 in the gut in the estimation set improved the fit further and was associated with drop in the OBJV (see figure 17). Drop in the objective value and corresponding improvement in the model fit with parameter estimation was reported in the tables below.
7. See S3 Table and S4 Table below for list parameters estimated in a step-wise manner and corresponding improvement in the model fit, in describing the data respectively.


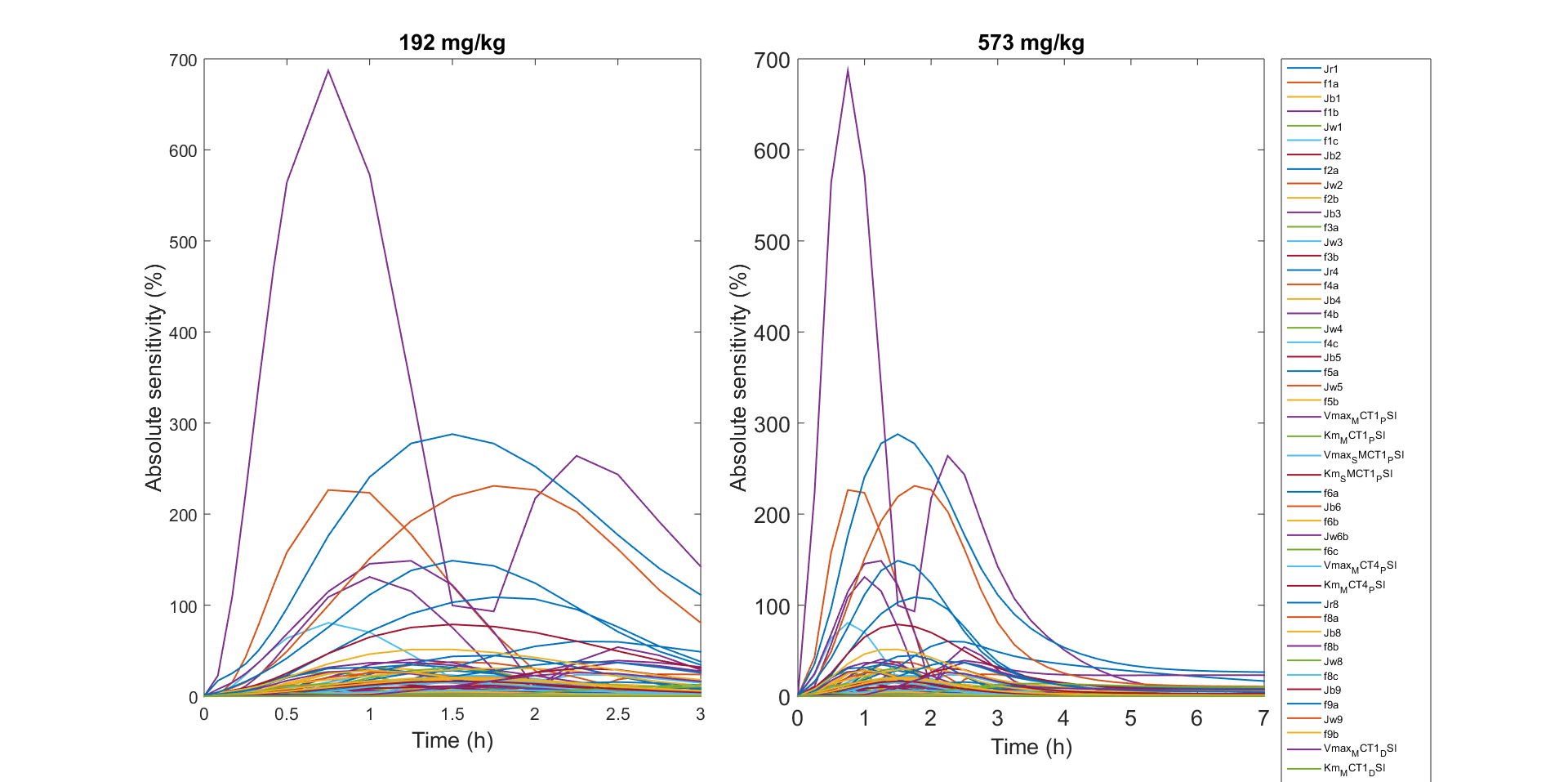


**S3 Fig. Parameter sensitivity in the systems model.** List of parameters and corresponding absolute sensitivity of parameters plotted over time.

**S3 Table. Parameter estimation steps in the systems model using maximum likelihood estimation method (**read Figure # in this table and see improvement in the model fit with parameter/s estimation in the corresponding figure # in **S4 Table)**

| S. No. | Parameter/s estimated | Initial Value | Estimated Value | OBJV Value | Figure # |
| --- | --- | --- | --- | --- | --- |
| 1 | f1a | 0.075 | 0.8258 | 712 | 1 |
| 2 | f1a and f1b | 0.075, 0.075 | 0.1884, 0.3291 | 423 | 2 |
| 3 | f1a, f1b and f4b | 0.075, 0.075, 0.05 | 0.1389, 0.3159, 0.00001 | 407 | 3 |
| 4 | f1a, f1b, f4b and f19b | 0.075, 0.075, 0.05 | 0.3439, 0.5630, 0.00701, 0.2149 | 352 | 4 |
| 5 | f1a, f1b, f4b, f19b and f24b | 0.075, 0.075, 0.05, 0.32 | 1.0004e-05, 0.48318, 1.0001e-05, 0.22127, 0.3719 | 346 | 5 |
| 6 | f1a, f1b, f4b, f19b, f24b and f29b | 0.075, 0.075, 0.05, 0.32, 0.2 | 2.05437, 99.9998, 1.04059e-05, 18.4772, 0.18238, 3.81558 | 465 | 6 |
| 7 | f1a, f1b, f4b, f19b, f24b, f29b and f30c | 0.075, 0.075, 0.05, 0.32, 0.2, 0.1 | 1.14428e-05, 99.9994, 1.04292e-05, 0.892687 1.00019e-05, 1.00016e-05, 5.08875 | 249 | 7 |
| 8 | f1a, f1b, f1c, f4b, f19b, f24b, f29b and f30c | 0.075, 0.075, 0.85, 0.05, 0.32, 0.2, 0.1 | 0.0000, 5.8617, 0.0000, 40.7361, 0.8953, 0.0000, 0.0000, 5.1194 | 249 | 8 |
| 9 | f1a, f1b, f4b, f8b, f19b, f24b, f29b and f30c | 0.075, 0.075, 0.05, 0.005, 0.32, 0.2, 0.1 | 1.72084e-05, 99.997, 1.21428e-05, 1.78478e-05, 0.892682, 1.00097e-05, 1.00082e-05, 5.08874 | 249 | 9 |
| 10 | f1a, f1b, f4b, f13a, f19b, f24b, f29b and f30c | 0.075, 0.075, 0.05, 0.05, 0.32, 0.2, 0.1 | 1.68029e-05, 37.7654, 1.05372e-05, 1.10609, 13.2627, 0.265909, 1.11212, 99.9999 | 422 | 10 |
| 11 | Jr1, Jb1, Jb4, Jb13, f19b, f24b, f29b and f30c | 4.5, 10, 3.5, 0.15, 0.32, 0.2, 0.1 | 100, 100, 1.01324e-05, 0.444182, 1.00008e-05, 1.00017e-05, 1.70294 | 168 | 11 |
| 12 | Jr1, Jb1, f1c, Jb4, Jb8, Jb13, Jb18, f19b, f24b, f29a, f29b and f30c | 4.5, 10, 0.85, 3.5, 2.5, 1, 10, 0.15, 0.32, 0.6, 0.2, 0.1 | 59.1811, 99.9934, 2.18385e-05, 61.9826, 51.4861, 5.83541, 0.000349733, 0.342019, 1.71144e-05, 4.02865, 8.03673e-05, 1.20119 | 102 | 12 |
| 13 | Jr1, Jb1, f1c, Jb4, Jb8, Jb9, f10a, Jb13, Jr17, Jb18, f19b, Jb22a, f24b, f29a, f29b and f30c | 4.5, 10, 0.85, 3.5, 2.5, 2.5, 0.5, 1, 4.5, 10, 0.15, 3.5, 0.32, 0.6, 0.2, 0.1 | 0, 0.2547, 99.2464, 2.3051, 0.0542, 1.1568, 0.0230, 0.0008, 7.7172, 49.7865, 14.6202, 0.2627, 26.4815, 0.0038, 1.5212, 0.0028, 1.2905 | -47.2 | 13 |
| 14 | Jr1, Jb1, Jr4, Jb4, Jb8, Jb9, f10a, Jb13, Jr17, f19a, Jr20a, Jb22a, Jb23a, f24a, f25a, f29b, f30c, eps1 and eps2 | 5, 10, 5, 5, 2.5, 2.5, 0.5, 1, 5, 0.8, 2.5, 3.5, 2.5, 0.5, 0.5, 0.2, 0.1 | 0.0985291, 45.8067, 0.0100347, 0.0253819, 0.589615, 11.7084, 0.000399619, 44.6615, 49.704 1.24172, 0.00475613, 99.218, 33.7535, 5.53277, 12.7344, 0.000805837, 2.00978, 0.0626519, 0.0999108 | -58 | 14 |
| 15 | Jr1, Jb1, Jr4, Jb4, Jb8, Jb9, f10a, Jb13, Jr17, f19a, Vmax_MCT1_Live, Jr20a, Jb22a, Jb23a, f24a, Vmax_MCT1_OT, f25a, f29b, f30c, eps1 and eps2 | 5, 10, 5, 5, 2.5, 2.5, 0.5, 1, 5, 0.8, 200, 2.5, 3.5, 2.5, 0.5, 250, 0.5, 0.2, 0.1 | 43.432401, 56.737057, 0.17276776, 0.20378367,8.9741107, 41.164949, 0.0033160288, 48.873422, 43.774738, 0.54161966, 780.94592, 1.0881908, 63.985581, 98.821897, 0.08527715, 1521.3929, 3.6042797, 0.000207429, 5.5303668, 0.067213394, 0.09523345 | -139 | 15 |
| 16 | Jr1, Jb1, Jb3, Jr4, Jb4, Jb5, f6a, Jb6, Jr8, Jb8, Jb9, f10a, Jb13, Jr17, f19a, Vmax_MCT1_Live, Jr20a, Jb22a, Jb22b, Jb23a, Jb23b, f24a, Vmax_MCT1_OT, f25a, f29b, f30c, eps1 and eps2 | 5, 10, 10, 5, 5, 3, 0.2, 3, 5, 2.5, 2.5, 0.5, 1, 5, 0.8, 200, 2.5, 3.5, 3.5, 2.5, 2.5, 0.5, 250, 0.5, 0.2, 0.1 | 29.84553, 42.89683, 2.024412, 0.02405532, 0.1299026, 3.97244, 0.06582878, 23.12405, 0.005103958, 0.570153, 3.688891, 1.481061, 57.72135, 79.61905, 0.5199499, 608.3329, 0.9160466, 14.88998, 30.83995, 94.53066, 30.8881, 0.251646, 711.7875, 22.04737, 0.01758391, 3.827028, 0.04446749, 0.09905792 | -153 | 16 |
| 17 | Jr1, Jb1, Jb3, Jr4, Jb4, Jb5, Vmax_MCT1_PSI, f6a, Jb6, Jr8, Jb8, Jb9, Vmax_MCT1_DSI, f10a, Jb13, Vmax_MCT1_Colon, Jr17, f19a, Vmax_MCT1_Liver, Jr20a, Jb22a, Jb22b, Jb23a, Jb23b, f24a, Vmax_MCT1_OT, f25a, f29b, f30c, eps1 and eps2 | 5, 10, 10, 5, 5, 3, 50, 0.2, 3, 5, 2.5, 2.5, 100, 0.5, 1, 25, 5, 0.8, 200, 2.5, 3.5, 3.5, 2.5, 2.5, 0.5, 250, 0.5, 0.2, 0.1 | 6.114138, 49.99681, 0.04449066, 20.95002, 0.0345074, 0.02175484, 14.74958, 0.001641403, 0.1324261, 0.01518031, 0.8067131, 14.74203, 189.816, 42.01082, 16.89422, 4.162906, 49.9938, 0.234214, 999.7959, 0.9712696, 44.46577, 1.066623, 49.96268, 48.93609, 0.4149686, 703.2612, 49.73017, 0.001021173, 22.41059, 0.0115671, 0.07133714 | -191 | 17 |

**S4 Table. Table showing improvement in the model fit in describing the data following parameter/s estimation by maximum likelihood estimation method**

| Parameter/s estimated – f1a (Figure 1) |
| --- |
|  |
| Parameter/s estimated – f1a and f1b (Figure 2) |
|  |
| Parameter/s estimated – f1a, f1b and f4b (Figure 3) |
|  |
| Parameter/s estimated – f1a, f1b, f4b and f19b (Figure 4) |
|  |
| Parameter/s estimated – f1a, f1b, f4b, f19b and f24b (Figure 5) |
|  |
| Parameter/s estimated – f1a, f1b, f4b, f19b, f24b and f29b (Figure 6) |
|  |
| Parameter/s estimated – f1a, f1b, f4b, f19b, f24b, f29b and f30c (Figure 7) |
|  |
| Parameter/s estimated – f1a, f1b, f1c, f4b, f19b, f24b, f29b and f30c (Figure 8) |
|  |
| Parameter/s estimated – f1a, f1b, f4b, f8b, f19b, f24b, f29b and f30c (Figure 9) |
|  |
| Parameter/s estimated – f1a, f1b, f4b, f13a, f19b, f24b, f29b and f30c (Figure 10) |
|  |
| Parameter/s estimated – Jr1, Jb1, Jb4, f19b, f24b, f29b and f30c (Figure 11) |
|  |
| Parameter/s estimated – Jr1, Jb1, f1c, Jb4, Jb8, Jb13, Jb18, f19b, f24b, f29a, f29b and f30c (Figure 12) |
|  |
| Parameter/s estimated – Jr1, Jb1, f1c, Jb4, Jb8, Jb9, f10a, Jb13, Jr17, Jb18, f19b, Jb22a, f24b, f29a, f29b and f30c (Figure 13) |
|  |
| Parameter/s estimated – Jr1, Jb1, Jr4, Jb4, Jb8, Jb9, f10a, Jb13, Jr17, f19a, Jr20a, Jb22a, Jb23a, f24a, f25a, f29a, and f30c (Figure 14) |
|  |
| Parameter/s estimated – Jr1, Jb1, Jr4, Jb4, Jb8, Jb9, f10a, Jb13, Jr17, f19a, Vmax_MCT1_Liver, Jr20a, Jb22a, Jb23a, f24a, Vmax_MCT1_OT, f25a, f29a, and f30c (Figure 15) |
|  |
| Parameter/s estimated – Jr1, Jb1, Jb3, Jr4, Jb4, Jb5, f6a, Jb6, Jr8, Jb8, Jb9, f10a, Jb13, Jr17, f19a, Vmax_MCT1_Liver, Jr20a, Jb22a, Jb22b, Jb23a, Jb23b, f24a, Vmax_MCT1_OT, f25a, f29a, and f30c (Figure 16) |
|  |
| Parameter/s estimated – Jr1, Jb1, Jb3, Jr4, Jb4, Jb5, Vmax_MCT1_PSI, f6a, Jb6, Jr8, Jb8, Jb9, Vmax_MCT1_DSI, f10a, Jb13, Vmax_MCT1_Colon, Jr17, f19a, Vmax_MCT1_Liver, Jr20a, Jb22a, Jb22b, Jb23a, Jb23b, f24a, Vmax_MCT1_OT, f25a, f29a, and f30c (Figure 17) |
|  |

**Ordinary differential equations (ODEs) of the final systems model:**

$$\frac{dA_{1}}{dt}=-A_{1}\cdot\left(f_{1a}\cdot J_{r1}+f_{1b}\cdot J_{b1}+f_{1c}\cdot J_{w1}\right)$$

$$\frac{dA_{2}}{dt}=A_{1}\cdot f_{1a}\cdot J_{r1}-A_{2}\cdot\left(f_{2a}\cdot J_{b2}+f_{2b}\cdot J_{w2}\right)$$

$$\frac{dA_{3}}{dt}=A_{1}\cdot f_{1a}\cdot J_{r1}-A_{3}\cdot\left(f_{3a}\cdot J_{b3}+f_{3b}\cdot J_{w3}\right)$$

$$\frac{dA_{4}}{dt}=A_{1}\cdot f_{1c}\cdot J_{w1}-A_{4}\cdot\left(f_{4a}\cdot J_{r4}+f_{4b}\cdot J_{b4}+f_{4c}\cdot J_{w4}\right)$$

$$\frac{dA_{5}}{dt}=A_{2}\cdot f_{2b}\cdot J_{w2}+A_{4}\cdot f_{4a}\cdot J_{r4}-A_{5}\cdot\left(f_{5a}\cdot J_{b5}+f_{5b}\cdot J_{w5}\right)$$

$$J_{w6a}=\left(\frac{V_{\max\_MCT_{1}\_PSI}}{\left(k_{m\_MCT_{1}\_PSI}+A_{6}\right)}\right)+\left(\frac{V_{\max\_SMCT_{1}\_PSI}}{\left(k_{m\_SMCT_{1}\_PSI}+A_{6}\right)}\right)$$

$$\frac{dA_{6}}{dt}=A_{3}\cdot f_{3b}\cdot J_{w3}+A_{4}\cdot f_{4a}\cdot J_{r4}-A_{6}\cdot\left(f_{6a}\cdot J_{w6a}+f_{6b}\cdot J_{b6}+f_{6c}\cdot J_{w6b}\right)$$

$$J_{b7}=\left(\frac{V_{\max\_MCT_{4}\_PSI}}{k_{m\_MCT_{4}\_PSI}+A_{7}}\right)$$

$$\frac{dA_{7}}{dt}=A_{6}\cdot f_{6a}\cdot J_{w6a}-A_{7}\cdot J_{b7}$$

$$\frac{dA_{8}}{dt}=A_{4}\cdot f_{4c}\cdot J_{w4}-A_{8}\left(f_{8a}\cdot J_{r8}+f_{8b}\cdot J_{b8}+f_{8c}\cdot J_{w8}\right)$$

$$\frac{dA_{9}}{dt}=A_{5}\cdot f_{5b}\cdot J_{w5}+A_{8}\cdot f_{8a}\cdot J_{r8}-A_{9}\cdot\left(f_{9a}\cdot J_{b9}+f_{9b}\cdot J_{w9}\right)$$

$$J_{w10a}=\left(\frac{V_{\max\_MCT_{1}\_DSI}}{\left(k_{m\_MCT_{1}\_DSI}+A_{10}\right)}\right)+\left(\frac{V_{\max\_SMCT_{1}\_DSI}}{\left(k_{m\_SMCT_{1}\_DSI}+A_{10}\right)}\right)$$

$$\begin{matrix} \frac{dA_{10}}{dt}=A_{6}\cdot f_{6c}\cdot J_{w6b}+A_{8}\cdot f_{8a}\cdot J_{r8}- \\ \text{ }A_{10}\cdot\left(f_{10a}\cdot J_{w10a}+f_{10b}\cdot J_{b10}+f_{10c}\cdot J_{w10b}\right) \end{matrix}$$

$$J_{b11}=\left(\frac{V_{\max\_MCT_{4}\_DSI}}{k_{m\_MCT_{4}\_DSI}+A_{11}}\right)$$

$$\frac{dA_{11}}{dt}=A_{10}\cdot f_{10a}\cdot J_{w10a}-A_{11}\cdot J_{b11}$$

$$\frac{dA_{12}}{dt}=A_{8}\cdot f_{8c}\cdot J_{w8}-A_{12}\cdot\left(f_{12a}\cdot J_{r12}+f_{12b}\cdot J_{b12}+f_{12c}\cdot J_{o12}\right)$$

$$\frac{dA_{13}}{dt}=A_{9}\cdot f_{9b}\cdot J_{w9}+A_{12}\cdot f_{12a}\cdot J_{r12}-A_{13}\cdot\left(f_{13a}\cdot J_{b13}+f_{13b}\cdot J_{o13}\right)$$

$$J_{w14}=\left(\frac{V_{\max\_MCT_{1}\_Colon}}{\left(k_{m\_MCT_{1}\_Colon}+A_{14}\right)}\right)+\left(\frac{V_{\max\_SMCT_{1}\_Colon}}{\left(k_{m\_SMCT_{1}\_Colon}+A_{14}\right)}\right)$$

$$\begin{matrix} \frac{dA_{14}}{dt}=A_{10}\cdot f_{10c}\cdot J_{w10b}+A_{12}\cdot f_{12a}\cdot J_{r12}- \\ \text{ }A_{14}\cdot\left(f_{14a}\cdot J_{w14}+f_{14b}\cdot J_{b14}+f_{14c}\cdot J_{o14}\right) \end{matrix}$$

$$J_{b15}=\left(\frac{V_{\max\_MCT_{4}\_Colon}}{k_{m\_MCT_{4}\_Colon}+A_{15}}\right)$$

$$\frac{dA_{15}}{dt}=A_{14}\cdot f_{14a}\cdot J_{w14}-A_{15}\cdot J_{b15}$$

$$J_{b16}=\left(\frac{V_{\max\_MCT_{1}\_Liver}}{k_{m\_MCT_{1}\_Liver}+A_{16}}\right)$$

$$\begin{matrix} \frac{dA_{16}}{dt}=A_{3}\cdot f_{3a}\cdot J_{b3}+A_{6}\cdot f_{6b}\cdot J_{b6}+A_{7}\cdot J_{b7}+A_{10}\cdot f_{10b}\cdot J_{b10}+ \\ \text{ }A_{11}\cdot J_{b11}+A_{14}\cdot f_{14b}\cdot J_{b14}+A_{15}\cdot J_{b15}-A_{16}\cdot J_{b16} \end{matrix}$$

$$\begin{matrix} \frac{dA_{17}}{dt}=A_{1}\cdot f_{1b}\cdot J_{b1}+A_{4}\cdot f_{4b}\cdot J_{b4}+A_{8}\cdot f_{8b}\cdot J_{b8}+A_{12}\cdot f_{12b}\cdot J_{b12}- \\ \text{ }A_{17}\cdot\left(f_{17a}\cdot J_{r17}+f_{17b}\cdot J_{b17}\right)+A_{22}\cdot f_{22b}\cdot J_{b22b} \end{matrix}$$

$$\begin{matrix} \frac{dA_{18}}{dt}=A_{2}\cdot f_{2a}\cdot J_{b2}+A_{5}\cdot f_{5a}\cdot J_{b5}+A_{9}\cdot f_{9a}\cdot J_{b9}+A_{13}\cdot f_{13a}\cdot J_{b13}+ \\ \text{ }A_{17}\cdot f_{17a}\cdot J_{r17}-A_{18}\cdot\left(f_{18a}\cdot J_{b18}+f_{18b}\cdot J_{r18}\right)+A_{23}\cdot f_{23b}\cdot J_{b23b} \end{matrix}$$

$$J_{b19}=V_{\max\_MCT_{1}\_Liver}\cdot\left(\frac{\left(\frac{1}{k_{m\_MCT_{1}\_Liver}}\right)}{1+\left(\frac{A_{19}}{k_{m\_MCT_{1}\_Liver}}\right)+\left(\frac{A_{20}}{k_{m\_MCT_{1}\_Liver\_AcAc}}\right)}\right)$$

$$\begin{matrix} \frac{dA_{19}}{dt}=A_{16}\cdot J_{b16}+A_{17}\cdot f_{17a}\cdot J_{r17}+A_{18}\cdot f_{18b}\cdot J_{r18}-\text{ } \\ \text{ }A_{19}\cdot\left(f_{19a}\cdot J_{b19}+f_{19b}\cdot J_{r19}\right)+A_{20}\cdot f_{20a}\cdot J_{r20a}+A_{24}\cdot f_{24b}\cdot J_{b24b} \end{matrix}$$

$$Rate_{in}=14.998$$

$$F_{effect}=1-\left(\left(\frac{I_{\max\_1}\cdot\left(A_{24}+A_{25}\right)}{IC_{50\_1}+\left(A_{24}+A_{25}\right)}\right)+\left(\frac{I_{\max\_2}\cdot A_{37}}{IC_{50\_2}+A_{37}}\right)\right)$$

$$J_{p20}=Rate_{in}\cdot F_{effect}$$

$$J_{b20}=V_{\max\_MCT_{1}\_Liver\_AcAc}\cdot\left(\frac{\left(\frac{1}{k_{m\_MCT_{1}\_Liver\_AcAc}}\right)}{1+\left(\frac{A_{19}}{k_{m\_MCT_{1}\_Liver}}\right)+\left(\frac{A_{20}}{k_{m\_MCT_{1}\_Liver\_AcAc}}\right)}\right)$$

$$\begin{matrix} \frac{dA_{20}}{dt}=A_{19}\cdot f_{19b}\cdot J_{r19}+J_{p20}-A_{20}\cdot\left(f_{20a}\cdot J_{r20a}+f_{20b}\cdot J_{r20b}+f_{20c}\cdot J_{b20}\right)+ \\ \text{ }A_{25}\cdot f_{25b}\cdot J_{b25b} \end{matrix}$$

$$\frac{dA_{21}}{dt}=A_{20}\cdot f_{20b}\cdot J_{r20b}-A_{21}\cdot J_{b21}+A_{26}\cdot f_{26b}\cdot J_{b26b}$$

$$\begin{matrix} \frac{dA_{22}}{dt}=A_{17}\cdot f_{17b}\cdot J_{b17}-A_{22}\cdot\left(f_{22a}\cdot J_{b22a}+f_{22b}\cdot J_{b22b}+f_{22c}\cdot J_{r22}\right)+ \\ \text{ }A_{27}\cdot f_{27a}\cdot J_{b27} \end{matrix}$$

$$\begin{matrix} \frac{dA_{23}}{dt}=A_{18}\cdot f_{18a}\cdot J_{b18}+A_{22}\cdot f_{22c}\cdot J_{r22}- \\ \text{ }A_{23}\cdot\left(f_{23a}\cdot J_{b23a}+f_{23b}\cdot J_{b23b}+f_{23c}\cdot J_{r23}\right)+A_{28}\cdot f_{28a}\cdot J_{b28} \end{matrix}$$

$$J_{b24a\_part1}=V_{\max\_MCT_{1}\_Tissues}\cdot\left(\frac{\left(\frac{1}{k_{m\_MCT_{1}\_Tissues}}\right)}{1+\left(\frac{A_{24}}{k_{m\_MCT_{1}\_Tissues}}\right)+\left(\frac{A_{25}}{k_{m\_MCT_{1}\_Tissues\_AcAc}}\right)}\right)$$

$$J_{b24a\_part2}=V_{\max\_MCT_{2}\_Tissues}\cdot\left(\frac{\left(\frac{1}{k_{m\_MCT_{2}\_Tissues}}\right)}{1+\left(\frac{A_{24}}{k_{m\_MCT_{2}\_Tissues}}\right)+\left(\frac{A_{25}}{k_{m\_MCT_{2}\_Tissues\_AcAc}}\right)}\right)$$

$$J_{b24a}=J_{b24a\_part1}+J_{b24a\_part2}$$

$$J_{b24b}=V_{\max\_MCT_{1}\_Liver}\cdot\left(\frac{\left(\frac{1}{k_{m\_MCT_{1}\_Liver}}\right)}{1+\left(\frac{A_{24}}{k_{m\_MCT_{1}\_Liver}}\right)+\left(\frac{A_{25}}{k_{m\_MCT_{1}\_Liver\_AcAc}}\right)}\right)$$

$$\begin{matrix} \frac{dA_{24}}{dt}=A_{19}\cdot f_{19a}\cdot J_{b19}+A_{22}\cdot f_{22c}\cdot J_{r22}+A_{23}\cdot f_{23c}\cdot J_{r23}- \\ \text{ }A_{24}\cdot\left(f_{24a}\cdot J_{b24a}+f_{24b}\cdot J_{b24b}\right)+A_{29}\cdot f_{29b}\cdot J_{b29} \end{matrix}$$

$$J_{b25a\_part1}=V_{\max\_MCT_{1}\_Tissues\_AcAc}\cdot\left(\frac{\left(\frac{1}{k_{m\_MCT_{1}\_Tissues\_AcAc}}\right)}{1+\left(\frac{A_{24}}{k_{m\_MCT_{1}\_Tissues}}\right)+\left(\frac{A_{25}}{k_{m\_MCT_{1}\_Tissues\_AcAc}}\right)}\right)$$

$$J_{b25a\_part2}=V_{\max\_MCT_{2}\_Tissues\_AcAc}\cdot\left(\frac{\left(\frac{1}{k_{m\_MCT_{2}\_Tissues\_AcAc}}\right)}{1+\left(\frac{A_{24}}{k_{m\_MCT_{2}\_Tissues}}\right)+\left(\frac{A_{25}}{k_{m\_MCT_{2}\_Tissues\_AcAc}}\right)}\right)$$

$$J_{b25a}=J_{b25a\_part1}+J_{b25a\_part2}$$

$$J_{b25b}=V_{\max\_MCT_{1}\_Liver\_AcAc}\cdot\left(\frac{\left(\frac{1}{k_{m\_MCT_{1}\_Liver\_AcAc}}\right)}{1+\left(\frac{A_{24}}{k_{m\_MCT_{1}\_Liver}}\right)+\left(\frac{A_{25}}{k_{m\_MCT_{1}\_Liver\_AcAc}}\right)}\right)$$

$$\begin{matrix} \frac{dA_{25}}{dt}=A_{20}\cdot f_{20c}\cdot J_{b20}-A_{25}\cdot\left(f_{25a}\cdot J_{b25a}+f_{25b}\cdot J_{b25b}+f_{25c}\cdot J_{r25}\right)+ \\ \text{ }A_{30}\cdot f_{30c}\cdot J_{b30} \end{matrix}$$

$$\begin{matrix} \frac{dA_{26}}{dt}=A_{21}\cdot J_{b21}+A_{25}\cdot f_{25c}\cdot J_{r25}- \\ \text{ }A_{26}\cdot\left(f_{26a}\cdot J_{b26a}+f_{26b}\cdot J_{b26b}\right)+A_{31}\cdot f_{31a}\cdot J_{b31} \end{matrix}$$

$$\frac{dA_{27}}{dt}=A_{22}\cdot f_{22a}\cdot J_{b22a}-A_{27}\cdot\left(f_{27a}\cdot J_{b27}+f_{27b}\cdot J_{o27}\right)$$

$$\frac{dA_{28}}{dt}=A_{23}\cdot f_{23a}\cdot J_{b23a}-A_{28}\cdot\left(f_{28a}\cdot J_{b28}+f_{28b}\cdot J_{o28}\right)$$

$$J_{b29\_part1}=V_{\max\_MCT_{1}\_Tissues}\cdot\left(\frac{\left(\frac{1}{k_{m\_MCT_{1}\_Tissues}}\right)}{1+\left(\frac{A_{29}}{k_{m\_MCT_{1}\_Tissues}}\right)+\left(\frac{A_{30}}{k_{m\_MCT_{1}\_Tissues\_AcAc}}\right)}\right)$$

$$J_{b29\_part2}=V_{\max\_MCT_{2}\_Tissues}\cdot\left(\frac{\left(\frac{1}{k_{m\_MCT_{2}\_Tissues}}\right)}{1+\left(\frac{A_{29}}{k_{m\_MCT_{2}\_Tissues}}\right)+\left(\frac{A_{30}}{k_{m\_MCT_{2}\_Tissues\_AcAc}}\right)}\right)$$

$$J_{b29}=J_{b29\_part1}+J_{b29\_part2}$$

$$\begin{matrix} \frac{dA_{29}}{dt}=A_{24}\cdot f_{24a}\cdot J_{b24a}-A_{29}\cdot\left(f_{29a}\cdot J_{r29}+f_{29b}\cdot J_{b29}+f_{29c}\cdot J_{o29}\right)+ \\ \text{ }A_{30}\cdot f_{30a}\cdot J_{r30a} \end{matrix}$$

$$J_{b30\_part1}=V_{\max\_MCT_{1}\_Tissues\_AcAc}\cdot\left(\frac{\left(\frac{1}{k_{m\_MCT_{1}\_Tissues\_AcAc}}\right)}{1+\left(\frac{A_{29}}{k_{m\_MCT_{1}\_Tissues}}\right)+\left(\frac{A_{30}}{k_{m\_MCT_{1}\_Tissues\_AcAc}}\right)}\right)$$

$$J_{b30\_part2}=V_{\max\_MCT_{2}\_Tissues\_AcAc}\cdot\left(\frac{\left(\frac{1}{k_{m\_MCT_{2}\_Tissues\_AcAc}}\right)}{1+\left(\frac{A_{29}}{k_{m\_MCT_{2}\_Tissues}}\right)+\left(\frac{A_{30}}{k_{m\_MCT_{2}\_Tissues\_AcAc}}\right)}\right)$$

$$J_{b30}=J_{b30\_part1}+J_{b30\_part2}$$

$$\begin{matrix} \frac{dA_{30}}{dt}=A_{25}\cdot f_{25a}\cdot J_{b25a}+A_{29}\cdot f_{29a}\cdot J_{r29}- \\ \text{ }A_{30}\cdot\left(f_{30a}\cdot J_{r30a}+f_{30b}\cdot J_{r30b}+f_{30c}\cdot J_{b30}+f_{30d}\cdot J_{o30}\right) \end{matrix}$$

$$\frac{dA_{31}}{dt}=A_{26}\cdot f_{26a}\cdot J_{b26a}-A_{31}\cdot\left(f_{31a}\cdot J_{b31}+f_{31b}\cdot J_{o31}\right)$$

$$\frac{dA_{32}}{dt}=A_{27}\cdot f_{27b}\cdot J_{o27}$$

$$\frac{dA_{33}}{dt}=A_{28}\cdot f_{28b}\cdot J_{o28}$$

$$\frac{dA_{34}}{dt}=A_{29}\cdot f_{29c}\cdot J_{o29}$$

$$\frac{dA_{35}}{dt}=A_{30}\cdot f_{30d}\cdot J_{o30}$$

$$\frac{dA_{36}}{dt}=A_{31}\cdot f_{31b}\cdot J_{o31}$$

$$\frac{dA_{37}}{dt}=Rate_{in}G-K_{out}G\cdot A_{37}$$

$$\frac{dA_{38}}{dt}=A_{12}\cdot f_{12c}\cdot J_{o12}$$

$$\frac{dA_{39}}{dt}=A_{13}\cdot f_{13b}\cdot J_{o13}$$

$$\frac{dA_{40}}{dt}=A_{14}\cdot f_{14c}\cdot J_{o14}$$

**References**

1. Ashy AA, Salleh M, Ardawi M. Glucose, glutamine, and ketone-body metabolism in human enterocytes. Metabolism. 1988;37(6):602-9.

2. Hanson PJ, Parsons DS. Factors affecting the utilization of ketone bodies and other substrates by rat jejunum: effects of fasting and of diabetes. J Physiol. 1978;278:55-67.

3. Deuticke B. Monocarboxylate transport in red blood cells: kinetics and chemical modification. Methods Enzymol. 1989;173:300-29.

4. Bruss ML. Chapter 4 - Lipids and Ketones. In: Bruss JJKWHL, editor. Clinical Biochemistry of Domestic Animals (Sixth Edition). San Diego: Academic Press; 2008. p. 81-115.

5. Cox PJ, Clarke K. Acute nutritional ketosis: implications for exercise performance and metabolism. Extrem Physiol Med. 2014;3:17-17.

6. Laffel L. Ketone bodies: a review of physiology, pathophysiology and application of monitoring to diabetes. Diabetes Metab Res Rev. 1999;15(6):412-26.

7. Wolfenden R, Lewis CA, Jr., Yuan Y. Kinetic challenges facing oxalate, malonate, acetoacetate, and oxaloacetate decarboxylases. J Am Chem Soc. 2011;133(15):5683-5.
