# Supplementary material for: An In Silico Knockout Model for Gastrointestinal Absorption Using a Systems Pharmacology Approach - Development and Application for Ketones
Source: PLoS One. 2016 Sep 29;11(9):e0163795. doi: 10.1371/journal.pone.0163795 (PMC5042539; doi:10.1371/journal.pone.0163795)
Supplement: S1 Table — (DOCX) [file pone.0163795.s004.docx]

**S1 Table. Initial condition/amount for states:**

| State | State | Amount (mmol) | Reference/Comment |
| --- | --- | --- | --- |
| 1 | Ester in upper proximal gut | Equivalent to dose of ester | - |
| 19 | BHB in liver | 1.018 | imputed to produce blood BHB of 0.1 mmol/L |
| 20 | AcAc in liver | 3.395 | imputed to produce blood AcAc of 0.1 mmol/L |
| 21 | Acetone in liver | 0.261 | assumed low concentration compared to BHB and AcAc |
| 24 | BHB in blood | 0.723 | imputed to produce blood BHB of 0.1 mmol/L, Davies et al 2003 |
| 25 | AcAc in blood | 0.742 | imputed to produce blood AcAc of 0.1 mmol/L, Davies et al 2003 |
| 26 | Acetone in blood | 0.456 | assumed low concentration compared to BHB and AcAc |
| 29 | BHB in tissues | 1.832 | imputed to produce blood BHB of 0.1 mmol/L |
| 30 | AcAc in tissues | 3.376 | imputed to produce blood AcAc of 0.1 mmol/L |
| 31 | Acetone in tissues | 0.374 | assumed low concentration compared to BHB and AcAc |
| 37 | Glucose in blood | 26.25 | Suckale et al 2008 |

All other states were assumed to have an initial amount of zero
