# Supplementary material for: An In Silico Knockout Model for Gastrointestinal Absorption Using a Systems Pharmacology Approach - Development and Application for Ketones
Source: PLoS One. 2016 Sep 29;11(9):e0163795. doi: 10.1371/journal.pone.0163795 (PMC5042539; doi:10.1371/journal.pone.0163795)
Supplement: S2 Table — (DOCX) [file pone.0163795.s005.docx]

**S2 Table. Parameter values used in the systems model:**

| S. No | Parameter | Assigned value | Unit | Reference/comment |
| --- | --- | --- | --- | --- |
| 1 | *J_r1_* | 6.11 | h^-1^ | Estimated |
| 2 | *f_1a_* | 0.075 | - | imputed assuming a little fraction of ester is metabolised in the stomach |
| 3 | *J_b1_* | 49.99 | h^-1^ | Estimated |
| 4 | *f_1b_* | 0.075 | - | imputed based on the assumption of limited absorption potential for paracellular transport |
| 5 | *J_w1_* | 2.31 | h^-1^ | Madsen et al., 1991 |
| 6 | *f_1c_* | 0.85 | - | assumed from parameter # 2, 4 |
| 7 | *J_b2_* | 10 | h^-1^ | imputed based on the absorption rate constant of BHB from the empirical model (Shivva et al., 2014) |
| 8 | *f_2a_* | 0.5 | - | imputed based on the fractional absorption of ethanol from stomach (Norberg et al., 2003) |
| 9 | *J_w2_* | 2.31 | h^-1^ | Madsen et al., 1991 |
| 10 | *f_2b_* | 0.5 | - | assumed from parameter # 8 |
| 11 | *J_b3_* | 0.0445 | h^-1^ | Estimated |
| 12 | *f_3a_* | 0.2 | - | imputed assuming smaller absorption potential in the stomach |
| 13 | *J_w3_* | 2.31 | h^-1^ | Madsen et al., 1991 |
| 14 | *f_3b_* | 0.8 | - | assumed from parameter # 12 |
| 15 | *J_r4_* | 20.95 | h^-1^ | Estimated |
| 16 | *f_4a_* | 0.3 | - | assumed greater metabolism of ester in the proximal small intestine |
| 17 | *J_b4_* | 0.0345 | h^-1^ | Estimated |
| 18 | *f_4b_* | 0.05 | - | imputed on the assumption of limited absorption potential for paracellular transport |
| 19 | *J_w4_* | 2.85 | h^-1^ | Madsen et al., 1991 |
| 20 | *f_4c_* | 0.65 | - | assumed from parameter # 16, 18 |
| 21 | *J_b5_* | 0.0217 | h^-1^ | Estimated |
| 22 | *f_5a_* | 0.2 | - | imputed based on the evidence of fractional absorption of ethanol from small intestine (Norberg et al., 2003) |
| 23 | *J_w5_* | 2.85 | h^-1^ | Madsen et al. 1991 |
| 24 | *f_5b_* | 0.8 | - | assumed from parameter # 22 |
| 25 | *V_max_MCT1_PSI_* | 14.75 | mmol. h^-1^ | estimated |
| 26 | *k_m_MCT1_PSI_* | 10.1 | mmol | Carpenter et al., 1994 |
| 27 | *V_max_SMCT1_PSI_* | 5.7 | mmol. h^-1^ | imputed based on the expression of MCT transporters in proximal gut (Gill et al., 2005) & surface area of small intestine (Kararli et al., 1995) |
| 28 | *k_m_SMCT1_PSI_* | 1.44 | mmol | Martin et al., 2006 |
| 29 | *f_6a_* | 0.00164 | - | estimated |
| 30 | *J_b6_* | 0.132 | h^-1^ | estimated |
| 31 | *f_6b_* | 0.05 | - | imputed based on the assumption that most of the BHB is ionised in the proximal small intestine |
| 32 | *J_w6b_* | 2.85 | h^-1^ | Madsen et al., 1991 |
| 33 | *f_6c_* | 0.75 | - | assumed from parameter # 29, 31 |
| 34 | *V_max_MCT4_PSI_* | 520 | mmol. h^-1^ | imputed based on the expression of MCT transporters in proximal gut (Gill et al., 2005) & surface area of small intestine (Kararli et al., 1995) |
| 35 | *k_m_MCT4_PSI_* | 130 | mmol | Manning Fox et al., 2000 |
| 36 | *J_r8_* | 0.0152 | h^-1^ | estimated |
| 37 | *f_8a_* | 0.5 | - | imputed assuming greater fraction of ester is metabolised in the distal small intestine |
| 38 | *J_b8_* | 0.806 | h^-1^ | estimated |
| 39 | *f_8b_* | 0.005 | - | imputed assuming low paracellular transport in the distal small intestine |
| 40 | *J_w8_* | 2.5 | h^-1^ | Madsen et al., 1991 |
| 41 | *f_8c_* | 0.495 | - | assumed from parameter # 37, 39 |
| 42 | *J_b9_* | 14.74 | h^-1^ | estimated |
| 43 | *f_9a_* | 0.3 | - | imputed based on the evidence for fractional absorption of ethanol from small intestine (Norberg et al., 2003) |
| 44 | *J_w9_* | 2.5 | h^-1^ | Madsen et al., 1991 |
| 45 | *f_9b_* | 0.7 | - | assumed from parameter # 43 |
| 46 | *V_max_MCT1_DSI_* | 189.82 | mmol. h^-1^ | estimated |
| 47 | *k_m_MCT1_DSI_* | 10.1 | mmol | Carpenter et al., 1994 |
| 48 | *V_max_SMCT1_DSI_* | 12 | mmol. h^-1^ | imputed based on the expression of MCT transporters in distal gut (Gill et al., 2005) & surface area of small intestine (Kararli et al., 1995) |
| 49 | *k_m_SMCT1_DSI_* | 1.44 | mmol | Martin et al., 2006 |
| 50 | *f_10a_* | 42.01 | - | estimated |
| 51 | *J_b10_* | 2.5 | h^-1^ | assumed based on the absorption rate constant of BHB from the empirical model (Shivva et al., 2014) |
| 52 | *f_10b_* | 0.05 | - | imputed assuming passive absorption is low in distal small intestine |
| 53 | *J_w10b_* | 2.5 | h^-1^ | Madsen et al., 1991 |
| 54 | *f_10c_* | 0.45 | - | assumed from parameter # 50, 52 |
| 55 | *V_max_MCT4_DSI_* | 860 | mmol. h^-1^ | imputed based on the expression of MCT transporters in distal gut (Gill et al., 2005) & surface area of small intestine (Kararli et al., 1995) |
| 56 | *k_m_MCT4_DSI_* | 130 | mmol | Manning Fox et al., 2000 |
| 57 | *J_r12_* | 4.5 | h^-1^ | imputed assuming ester breakdown is rapid in colon |
| 58 | *f_12a_* | 0.75 | - | assumed greater hydrolysis of ester in colon due to long transit time |
| 59 | *J_b1_2* | 1 | h^-1^ | imputed low absorption rate due to tight junctions in colon |
| 60 | *f_12b_* | 0.005 | - | imputed low fractional absorption due to tight junctions in colon |
| 61 | *J_o12_* | 0.17 | h^-1^ | Madsen et al., 1991 |
| 62 | *f_12c_* | 0.245 | - | assumed from parameter # 58, 60 |
| 63 | *J_b13_* | 16.89 | h^-1^ | estimated |
| 64 | *f_13a_* | 0.05 | - | imputed low fractional absorption due to tight junctions in colon |
| 65 | *J_o13_* | 0.17 | h^-1^ | Madsen et al., 1991 |
| 66 | *f_13b_* | 0.95 | - | assumed from parameter # 64 |
| 67 | *V_max_MCT1_Colon_* | 4.16 | mmol. h^-1^ | estimated |
| 68 | *k_m_MCT1_Colon_* | 10.1 | mmol | Carpenter et al., 1994 |
| 69 | *V_max_SMCT1_Colon_* | 2.8 | mmol. h^-1^ | imputed based on the expression of MCT transporters in distal gut (Gill et al., 2005) & surface area of colon  (Kararli et al., 1995) |
| 70 | *k_m_SMCT1_Colon_* | 1.44 | mmol | Martin et al., 2006 |
| 71 | *f_14a_* | 0.75 | - | assumed greater fractional absorption due to high expression of transport proteins and long transit time in colon |
| 72 | *J_b14_* | 1 | h^-1^ | imputed low absorption rate due to tight junctions in colon |
| 73 | *f_14b_* | 0.005 | - | imputed low fractional absorption due to tight junctions in colon |
| 74 | *J_o14_* | 0.17 | h^-1^ | Madsen et al., 1991 |
| 75 | *f_14cc_* | 0.245 | - | assumed from parameter # 71, 73 |
| 76 | *V_max_MCT4_Colon_* | 350 | mmol. h^-1^ | imputed based on the expression of MCT transporters in distal gut (Gill et al., 2005) & surface area of colon  (Kararli et al., 1995) |
| 77 | *k_m_MCT4_Colon_* | 130 | mmol | Manning Fox et al., 2000 |
| 78 | *J_r17_* | 49.99 | h^-1^ | estimated |
| 79 | *f_17a_* | 0.7 | - | greater fraction assumed for hydrolysis of ester in the liver |
| 80 | *J_b17_* | 10 | h^-1^ | imputed based on venous blood flow rate from the liver via hepatic vein |
| 81 | *f_17b_* | 0.3 | - | assumed from parameter # 79 |
| 82 | *J_b18_* | 10 | h^-1^ | imputed based on venous blood flow rate from the liver via hepatic vein |
| 83 | *f_18a_* | 0.3 | - | imputed assuming greater fraction of butanediol metabolised in the liver |
| 84 | *J_r18_* | 6.5 | h^-1^ | imputed assuming that butanediol metabolism is rapid in the liver |
| 85 | *f_18b_* | 0.7 | - | assumed from parameter # 83 |
| 86 | *f_19a_* | 0.23 | - | estimated |
| 87 | *J_r19_* | 2.5 | h^-1^ | imputed assuming that interconversion of BHB and AcAc in the liver is rapid |
| 88 | *f_19b_* | 0.15 | - | assumed from parameter # 86 |
| 89 | *V_max_MCT1_Liver_* | 999.7 | mmol. h^-1^ | estimated |
| 90 | *k_m_MCT1_Liver_* | 10.1 | mmol | Carpenter et al., 1994 |
| 91 | *J_r20a_* | 0.97 | h^-1^ | estimated |
| 92 | *f_20a_* | 0.7 | - | imputed based on the evidence that the liver do not consume ketones |
| 93 | *J_r20b_* | 3.5 | h^-1^ | imputed based on the assumption that decarboxylation of AcAc is rapid and a spontaneous process |
| 94 | *f_20b_* | 0.05 | - | imputed based on assumption that decarboxylation has limited scope in the liver |
| 95 | *f_20c_* | 0.25 | - | assumed from parameter # 92, 94 |
| 96 | *V_max_MCT1_Liver_AcAc_* | 105 | mmol. h^-1^ | scaled from the *in vitro* data  (Meredith et al. 2008) |
| 97 | *k_m_MCT1_Liver_AcAc_* | 5.5 | mmol | Carpenter et al., 1994 |
| 98 | *I_max_1_* | 0.45 | mmol. h^-1^ | imputed based on the knowledge from empirical PK model for BHB (Shivva et al., 2014) |
| 99 | *IC_50_1_* | 4.5 | mmol | imputed based on the knowledge from empirical PK model for BHB  (Shivva et al., 2014) |
| 100 | *J_b21_* | 3.5 | h^-1^ | imputed based on venous blood flow rate from the liver via hepatic vein |
| 101 | *J_r22_* | 4.5 | h^-1^ | imputed assuming that ester metabolism is rapid in blood |
| 102 | *f_22a_* | 0.5 | - | imputed assuming that most of the ester is metabolised in blood |
| 103 | *J_b22a_* | 44.46 | h^-1^ | estimated |
| 104 | *f_22b_* | 0.05 | - | imputed assuming that most of the ester is metabolised in blood |
| 105 | *J_b22b_* | 1.07 | h^-1^ | estimated |
| 106 | *f_22c_* | 0.45 | - | assumed from parameter # 102, 104 |
| 107 | *J_b23a_* | 49.96 | h^-1^ | estimated |
| 108 | *f_23a_* | 0.3 | - | imputed assuming greater fraction of butanediol is metabolised in blood |
| 109 | *J_b23b_* | 48.94 | h^-1^ | estimated |
| 110 | *f_23b_* | 0.05 | - | imputed assuming greater fraction of butanediol is metabolised in blood |
| 111 | *J_r23_* | 6.5 | h^-1^ | imputed assuming rapid metabolism for butanediol in blood |
| 112 | *f_23c_* | 0.65 | - | assumed from parameter # 108, 110 |
| 113 | *f_24a_* | 0.415 | - | estimated |
| 114 | *f_24b_* | 0.32 | - | assumed from parameter # 113 |
| 115 | *V_max_MCT1_OT_* | 703.26 | mmol. h^-1^ | estimated |
| 116 | *k_m_MCT1_OT_* | 10.1 | mmol | Carpenter et al. 1994 |
| 117 | *V_max_MCT2_OT_* | 12.2 | mmol. h^-1^ | imputed based on the evidence that MCT2 is a low capacity transporter (Meredith et al. 2008) |
| 118 | *k_m_MCT2_OT_* | 1.2 | mmol | Bröer et al., 1999 |
| 119 | *f_25a_* | 49.73 | - | estimated |
| 120 | *f_25b_* | 0.05 | - | imputed on the evidence that only little/no fraction of ketones are consumed in the liver |
| 121 | *J_r25_* | 4.5 | h^-1^ | imputed assuming that decarboxylation of AcAc in blood is rapid and spontaneous |
| 122 | *f_25c_* | 0.3 | - | assumed from parameter # 119, 120 |
| 123 | *V_max_MCT1_OT_AcAc_* | 165 | mmol. h^-1^ | imputed based on the evidence that MCT1 is a high capacity transporter (Meredith et al. 2008) |
| 124 | *k_m_MCT1_OT_AcAc_* | 5.5 | mmol | Carpenter et al., 1994 |
| 125 | *V_max_MCT2_OT_AcAc_* | 8.5 | mmol. h^-1^ | imputed based on the evidence that MCT2 is a low capacity transporter (Meredith et al. 2008) |
| 126 | *k_m_MCT2_OT_AcAc_* | 0.8 | mmol | Bröer et al., 1999 |
| 127 | *J_b26a_* | 4.5 | h^-1^ | imputed assuming rapid distribution and excretion of acetone to lungs |
| 128 | *f_26a_* | 0.8 | - | imputed on the assumption of huge capacity for lungs in expelling acetone in the breath |
| 129 | *J_b26b_* | 3.5 | h^-1^ | imputed assuming rapid distribution for acetone to liver from blood |
| 130 | *f_26b_* | 0.2 | - | assumed from parameter # 128 |
| 131 | *J_b27_* | 3.5 | h^-1^ | imputed based on assumption of rapid transport for ester out of tissues |
| 132 | *f_27a_* | 0.005 | - | imputed on the assumption that only minor fraction of ester is reabsorbed from the kidneys |
| 133 | *J_o27_* | 2.75 | h^-1^ | imputed on the assumption that excretion of ester is a rapid process via the kidneys |
| 134 | *f_27b_* | 0.995 | - | assumed from parameter # 132 |
| 135 | *J_b28_* | 2.5 | h^-1^ | imputed based on assumption of rapid transport for butanediol out of tissues |
| 136 | *f_28a_* | 0.005 | - | imputed on the assumption that only minor fraction of butanediol is reabsorbed from the kidneys |
| 137 | *J_o28_* | 3.75 | h^-1^ | imputed on the assumption that excretion of butanediol is a rapid process via the kidneys |
| 138 | *f_28b_* | 0.995 | - | assumed from parameter # 136 |
| 139 | *J_r29_* | 4.5 | h^-1^ | imputed on the evidence that metabolism of BHB is rapid in tissues (Robinson et al., 1980) |
| 140 | *f_29a_* | 0.6 | - | imputed assuming major fraction of BHB is converted to AcAc in tissues to be used as energy source |
| 141 | *f_29b_* | 0.00102 | - | estimated |
| 142 | *J_o29_* | 3.75 | h^-1^ | imputed on the assumption that excretion of BHB is a rapid process via the kidneys |
| 143 | *f_29c_* | 0.2 | - | assumed from parameter # 140, 141 |
| 144 | *J_r30a_* | 4.5 | h^-1^ | imputed on the assumption of rapid reconversion of AcAc to BHB in tissues (Robinson et al., 1980) |
| 145 | *f_30a_* | 0.05 | - | imputed assuming minor fraction of AcAc is reconverted to BHB in tissues to be used as energy source |
| 146 | *J_r30b_* | 4.5 | h^-1^ | imputed assuming rapid process for utilization of AcAc in Krebs cycle |
| 147 | *f_30b_* | 0.5 | - | imputed assuming major fraction of AcAc is used as energy source in tissues |
| 148 | *f_30c_* | 22.41 | - | estimated |
| 149 | *J_o30_* | 3.75 | h^-1^ | guess based on the assumption that glomerular filtration and tubular secretion in kidneys for BHB is a rapid process |
| 150 | *f_30d_* | 0.35 | - | assumed from parameter # 145, 147, 148 |
| 151 | *J_b31_* | 2.5 | h^-1^ | imputed with an assumption of low redistribution of acetone into blood |
| 152 | *f_31a_* | 0.05 | - | imputed on the assumption that only minor fraction of acetone is redistributed into blood from lungs |
| 153 | *J_o31_* | 4.5 | h^-1^ | imputed on the assumption that expulsion of acetone is rapid from lungs |
| 154 | *f_31b_* | 0.95 | - | assumed from parameter # 152 |
| 155 | *V_17_* | 1.7 | L | Davies et al., 1993 |
| 156 | *V_18_* | 1.7 | L | Davies et al., 1993 |
| 157 | *V_19_* | 1.7 | L | Davies et al., 1993 |
| 158 | *V_20_* | 1.7 | L | Davies et al., 1993 |
| 159 | *V_21_* | 1.7 | L | Davies et al., 1993 |
| 160 | *V_22_* | 5.2 | L | Davies et al., 1993 |
| 161 | *V_23_* | 5.2 | L | Davies et al., 1993 |
| 162 | *V_24_* | 5.2 | L | Davies et al., 1993 |
| 163 | *V_25_* | 5.2 | L | Davies et al., 1993 |
| 164 | *V_26_* | 5.2 | L | Davies et al., 1993 |
| 165 | *V_27_* | 5.6 | L | Davies et al., 1993 |
| 166 | *V_28_* | 5.6 | L | Davies et al., 1993 |
| 167 | *V_29_* | 5.6 | L | Davies et al., 1993 |
| 168 | *V_30_* | 5.6 | L | Davies et al., 1993 |
| 169 | *V_31_* | 5.6 | L | Davies et al., 1993 |
| 170 | *Rate_inG_* | 82.95 | mmol.h^-1^ | Aronoff et al., 2004 |
| 171 | *k_outG_* | 3.16 | h^-1^ | imputed based on rate of appearance and basal concentrations of glucose  (Aronoff et al., 2004) |
| 172 | *I_max-2_* | 0.35 | mmol.h^-1^ | imputed assuming endogenous ketone production is massively supressed under normal conditions of blood glucose |
| 173 | *IC_50-2_* | 22.5 | mmol | imputed assuming endogenous ketone production is massively supressed under normal conditions of blood glucose |
